# Supplementary material for: A partially self-regenerating synthetic cell
Source: Nat Commun. 2020 Dec 11;11:6340. doi: 10.1038/s41467-020-20180-6 (PMC7733450; doi:10.1038/s41467-020-20180-6)
Supplement: Supplementary file 1 — Supplementary Information [file 41467_2020_20180_MOESM1_ESM.pdf]

Supplementary Information:
A partially self-regenerating synthetic cell

Barbora Lavickova[1], Nadanai Laohakunakorn[2], and Sebastian J. Maerkl[*1]

[1]Institute of Bioengineering, School of Engineering, École Polytechnique Fédérale de Lausanne, Lausanne, Switzerland

[2]Institute of Quantitative Biology, Biochemistry, and Biotechnology, School of Biological Sciences, University of Edinburgh, Edinburgh, United Kingdom

[*]Correspondence: sebastian.maerkl@epfl.ch

Supplementary Information

Supplementary Text

Supplementary Figures 1 to 24

Supplementary Tables 1 to 8

Modeling

Minimal resource-dependent TX-TL model

While cell-free transcription and translation can be described at varying levels of granularity [1, 2, 3, 4, 5, 6, 7, 8], here we chose to model the processes at the most coarse-grained level using coupled ordinary differential equations (ODEs). This model can be easily extended to incorporate more complex effects, but the aim here was to show a minimal mechanism which qualitatively captures the observed experimental effects.

The model consists of simultaneous transcription and translation of GFP and T7 RNAP, which consumes a single resource species R. This species is a lumped representation of NTPs which are consumed during transcription, and ATP, GTP, and aminoacyl tRNAs which are consumed during translation. We model the transcription rate by a parameter α, linearly dependent on DNA and T7 RNAP concentration, and modulated by the availability of resources using a Hill function $R/(R+K)$. Likewise, translation proceeds at a rate β, which is linearly dependent on mRNA concentration, and is modulated by the same Hill function for resource dependence. The rate of consumption of R is equal to the summed transcription and translation rates. The complete model consisting of seven ODEs and three parameters, is shown below.

$$\dot{R} = -\dot{m_T} - \dot{m_G} - \dot{p_T} - \dot{p_G} \tag{1}$$

$$\dot{d_T} = 0 \tag{2}$$

$$\dot{d_G} = 0 \tag{3}$$

$$\dot{m_T} = \alpha \frac{R}{R+K} d_T p_T \tag{4}$$

$$\dot{m_G} = \alpha \frac{R}{R+K} d_G p_T \tag{5}$$

$$\dot{p_T} = \beta \frac{R}{R+K} m_T \tag{6}$$

$$\dot{p_G} = \beta \frac{R}{R+K} m_G \tag{7}$$

DNA, mRNA, and protein concentrations are denoted by d, m, and p, and the subscripts T and G refer to T7 RNAP and eGFP respectively. The model was implemented in Julia 1.4.2 and solved using the `DifferentialEquations.jl` package. All code is available on github.

Chemostat simulation

During chemostat operation, concentrations of species in the cell-free reaction are periodically adjusted. All components are diluted at a specific dilution fraction, while certain components (proteins, ribosomes, energy solution, and DNA) are replenished. This can be captured in the model by explicitly including the dilution steps. In Julia this is achieved by implementing callbacks which modify the concentrations at specified time points while solving the ODEs. More detail can be found in the documentation for the code.

Simulating the chemostat leads to a sawtooth-like behaviour as eGFP is diluted, and subsequently produced between dilution steps, as shown by the green curve in Supplementary Fig. 9. In the real experiment, images are taken immediately before each dilution step, and thus the data appear smooth: this is shown by the dashed black curve in Supplementary Fig. 9.

At each dilution step in the model, all species' concentrations are reduced by a fixed dilution fraction, while the concentrations of certain species are refreshed by addition of a fraction of those species at their initial concentrations:

$$c_{i+1} = c_i(1 - \gamma) + \gamma c_0 \tag{8}$$

The dilution fraction γ was set to 20%, and the periodicity of dilution to 15 minutes, corresponding to experimental values. Each *in silico* experiment contained three stages: 1.) kick-start, 2.) self-regeneration, and 3.) washout. The species were replenished as indicated in Supplementary Table 5. The negative control corresponded to a self-regeneration experiment with $d_T = 0$.

Model design and parameters

Since our overall goal was to capture qualitative rather than quantitative behaviour, we used effective parameters and arbitrary units to describe system dynamics, which were combined with physical time values and experimental chemostat operation parameters. Nevertheless, initial parameter selection was guided by relative magnitudes of various parameters. In particular, the resource saturation term K is typically several orders of magnitude less than the initial resource concentration [9], and cell-free translation rates are typically an order of magnitude slower than transcription [10]. The initial parameter set was manually chosen to reflect experimentally-observed behaviour; parameter scans were then conducted to test the robustness of model behaviour on parameter variations, as discussed below.

We have assumed equal resource consumption for transcription and translation, which is motivated by the fact that translation consumes $2N$ GTP and N ATP to synthesise a polypeptide of length N (accounting for aminoacylation), while transcription consumes on average $3N/4$ ATP and $3N/4$ GTP (as well as UTP and CTP), which is the same as translation to within an order of magnitude.

Elucidation of model behaviour

In order to elucidate the origins of the observed behaviour, we can inspect protein, mRNA, and resource levels, as shown in Supplementary Fig. 10. Because translation rate is the product of mRNA concentration and the resource dependence term $R/(R+K)$, high levels of translation require both to be present. Let us consider the self-regeneration phase (4–16h). At low concentrations of d_T, resource levels are high; however mRNA concentrations are low, and thus overall the translation rate of eGFP is low. In the converse situation at high d_T, mRNA levels are high, but resources are low, leading once again to low eGFP production. It is only at intermediate concentrations of d_T where eGFP production is high when there is a small but nonzero amount of resource availability, as well as an intermediate level of mRNA present. The model thus predicts that the production of eGFP is determined by a trade-off between resource availability and mRNA concentration.

In order to further interrogate the model, we can look at transcription and translation rates. These can be determined from the model by evaluating the derivatives directly from the ODEs. As the system is periodically diluted, the rates are also periodically modulated. An example is shown in Supplementary Fig. 11A (grey line), from which we can calculate the average rate (green line). Here we observe that the translation rate of GFP in the positive control experiment varies from a high value to zero in every cycle. This is due to resources being completely depleted in each cycle, as shown in Supplementary Fig. 11B. The average rates of transcription and translation of GFP and T7 RNAP are shown in Supplementary Fig. 12, where we again observe that increasing d_T increases average transcription rates (Supplementary Fig. 12B), but decreases translation rates (Supplementary Fig. 12A).

In our model the consumption of resources is directly equal to the summed transcription and translation rates. Thus we can determine the allocation of resources between different model processes, as shown in Supplementary Fig. 13. We observe that at the onset of the self-regeneration phase, transcriptional resource consumption decreases while translational consumption increases

(Fig 13A and B), which forms one part of our hypothesis to explain the increase in eGFP production over the positive control. Supplementary Fig. 13E and F show that as T7 RNAP is washed out at late times, resource allocation tends to 100% translation, and this accounts for the 'bump' in eGFP production in the washout phase.

We can also carry out parameter variations, shown in Supplementary Fig. 14 as a contour map of the variation of the parameter of interest against a T7 DNA titration. Here we show the model predictions for two quantities: the ratio of self-regeneration to positive control (SR/PC) at 15h, as shown in the main text, and the overall eGFP production at 15h, which is a measure of the productivity of the self-regeneration process. These results yield further insights into the mechanisms of the model; of relevance is the observation that both eGFP production and the ratio SR/PC exhibits an optimum with respect to T7 RNAP DNA, and the position of the optimum is only significantly affected by transcription and translation rates: increasing these rates shifts the optimum to lower values. The optimum is otherwise relatively robust; in particular, the position of the SR/PC optimum is insensitive to d_G (shown in more detail in Supplementary Fig. 15). An important observation is that the the ratio SR/PC contains an optimum for high values of T7 DNA and small values of the initial resource concentration R_0. The interpretation of this is that the model predicts that high SR/PC ratios are achieved when the resources become scarce.

In summary, analysis of the single resource-dependent model behaviour leads to two main conclusions:

1. eGFP production depends on a trade-off between resource availability and mRNA concentration. As d_T is increased, eGFP production therefore exhibits a maximum.

2. For intermediate concentrations, eGFP production is higher than the positive control during the self-regeneration phase. This is accounted for by a reallocation of resources from transcription to translation during the transition between kick-start and self-regeneration, and by an overall resource-limited condition.

Sufficiency of model mechanism

We would like to understand whether the resource-dependent model is necessary and sufficient to explain our observations. Therefore we developed a second model, whose transcriptional and translational activities do not depend on any resource. This 'resource-independent' model instead

contains TX and TL rates which decrease exponentially over time, with a fixed decay constant λ, which represents a resource-independent inactivation of cell-free protein synthesis. Such effects are also observed experimentally [2]. This model can be written as follows:

$$\dot{d_T} = 0 \tag{9}$$

$$\dot{d_G} = 0 \tag{10}$$

$$\dot{m_T} = \alpha \exp(-\lambda t) d_T p_T \tag{11}$$

$$\dot{m_G} = \alpha \exp(-\lambda t) d_G p_T \tag{12}$$

$$\dot{p_T} = \beta \exp(-\lambda t) \frac{m_T}{m_T + m_G + K_{TL}} \tag{13}$$

$$\dot{p_G} = \beta \exp(-\lambda t) \frac{m_G}{m_T + m_G + K_{TL}} \tag{14}$$

Here, we model translation as saturating at high total mRNA concentrations, with a Hill function and a saturation constant K_{TL}. This is a typical way of taking into account translational loading effects [11]. We observe that this model can also qualitatively capture some of the experimental observations. Supplementary Fig. 16A shows the time courses and SR/PC ratio plot of the single-resource model. Supplementary Fig. 16B shows the same for the resource-independent model, which again captures the optimum in SR/PC ratio as a function of T7 RNAP DNA. The model exhibits the three features of decaying eGFP production at low d_T, high eGFP (potentially above the positive control level) at intermediate d_T, and low eGFP production followed by a peak during washout at high d_T.

The explanation of a maximum in eGFP production as a function of T7 RNAP DNA, is different from in the single-resource model. Here, at low d_T, the concentration of m_G is low, leading to low translation rates. At high d_T, the concentration of m_T is high, loading the translational machinery and again leading to low translation rates. At intermediate concentrations, where mRNA concentrations are high but before translational loading effects set in, we observe a maximum eGFP production.

Despite the different mechanism, there is a crucial similarity between this and the resource-dependent model: both involve coupling of the expression of eGFP and T7 RNAP. In the resource-dependent model, this is through a shared resource term, and in the resource-independent model, this is through a shared translational term. To demonstrate this, the coupling term can be artificially removed in the resource-independent model, allowing each protein to be translated independently. This leads to Supplementary Fig. 16C, where the ratio SR/PC monotonically increases

with increasing T7 RNAP template, and no maximum is observed.

A second feature of both models is the striking increase of GFP above positive control levels (SR/PC> 1), for intermediate T7 RNAP template concentrations. These again result from two different mechanisms. In the resource-dependent model, analysis from the previous section shows that this is due to release of resources, under resource-limited conditions.

In the resource-independent model, T7 RNAP concentration is low in the kick-start phase. Thus any increase in T7 RNAP DNA template will increase T7 RNAP concentration, leading to greater transcription and translation, with no incurred costs. As long as translational capacity is not loaded, this effect can increase GFP over the positive control level. The increase of GFP is thus due to the activity of extra T7 RNAP in the system. However, the increase begins immediately in the kick-start phase, and is maintained throughout self-regeneration.

The explanation for these mechanisms can be tested *in silico*: in the first case, increasing the availability of resources should alleviate the resource constraint, and decrease the SR/PC ratio. This is observed in the parameter study shown in Supplementary Fig. 14. In the second case, increasing the initial T7 RNAP concentration should decrease the effect of any additional T7 RNAP produced. This is also observed in a similar parameter exploration, shown in Supplementary Fig. 17.

In reality, it is likely that both mechanisms are at play. While the PURE system is known to be resource-limited under certain conditions [12], some lysate-based systems exhibit resource-independent deactivation [13]. Since a model which simultaneously takes both effects into account is likely to be more general, we tested a combined model, whose results are shown in Supplementary Fig. 16D and 18. While this model also successfully captures experimental observations, it is less robust than the simpler models, requiring fine-tuning of parameters. Experimentally, since we observe an increase in GFP after self-regeneration begins, and not immediately from the beginning of the kick-start phase, it is likely that under our experimental conditions, the resource limitation is a more dominant effect.

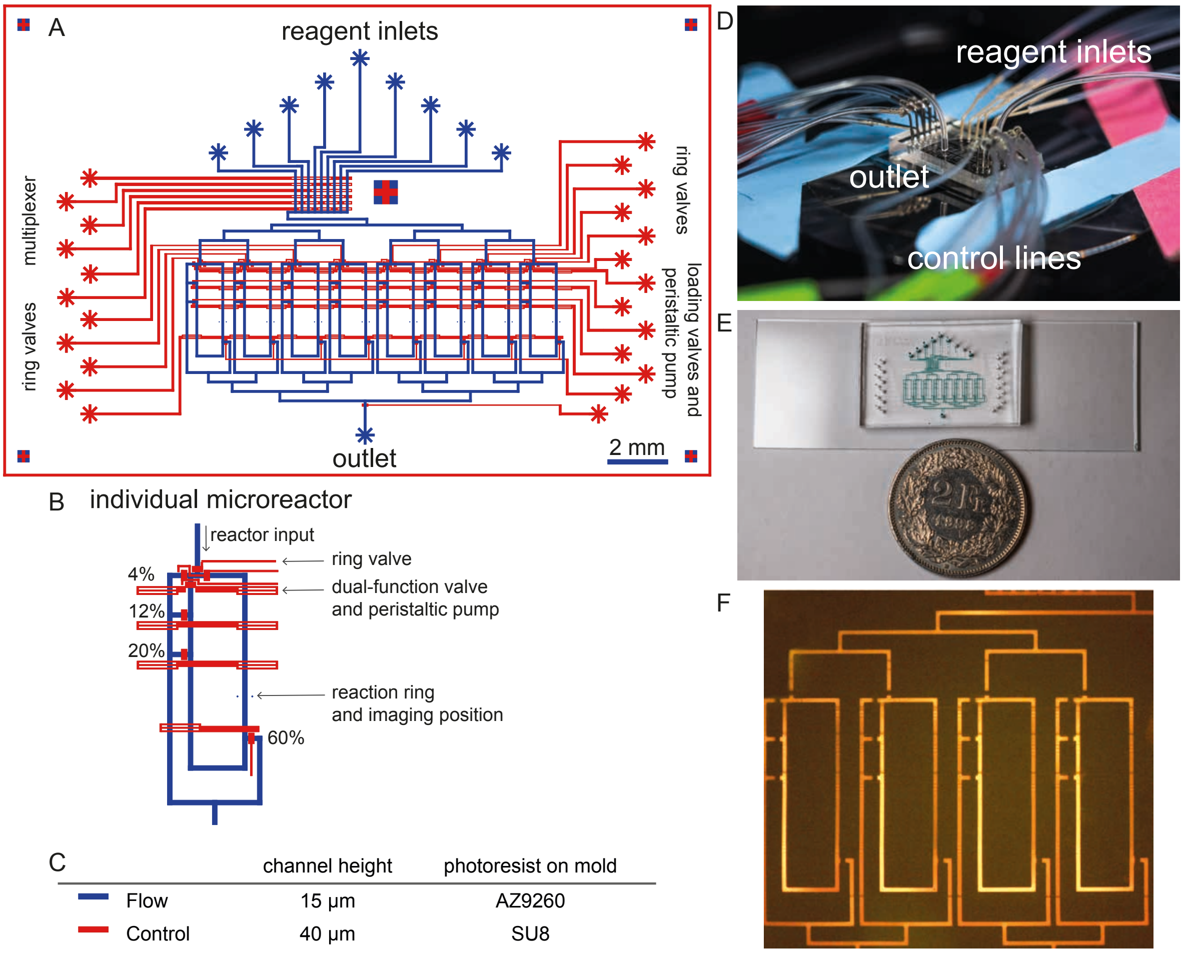

	channel height	photoresist on mold
Flow	15 µm	AZ9260
Control	40 µm	SU8

Supplementary Figure 1: **Microfluidic device design:**

(A) Design schematic of the microfluidic device. The control layer is shown in red and the flow layer in blue. The device contains eight individually addressable chemostat reactors. **(B)** Close-up of a microfluidic reactor. Each reactor has four outlets corresponding to four different dilution fractions. Four control lines serve dual-functions as valves and peristaltic pump. The width of a flow channel is 100μm. **(C)** Table of channel heights and corresponding photoresists used in mold fabrication. **(D)** Image of microfluidic chip connected to the control lines, reagent inputs and outlet. **(E)** Image of microfluidic chip, flow channels are filled with blue dye for visualization. **(F)** Microscope images of four microreactors. Control channels are filled with fluorescence dye for visualization.

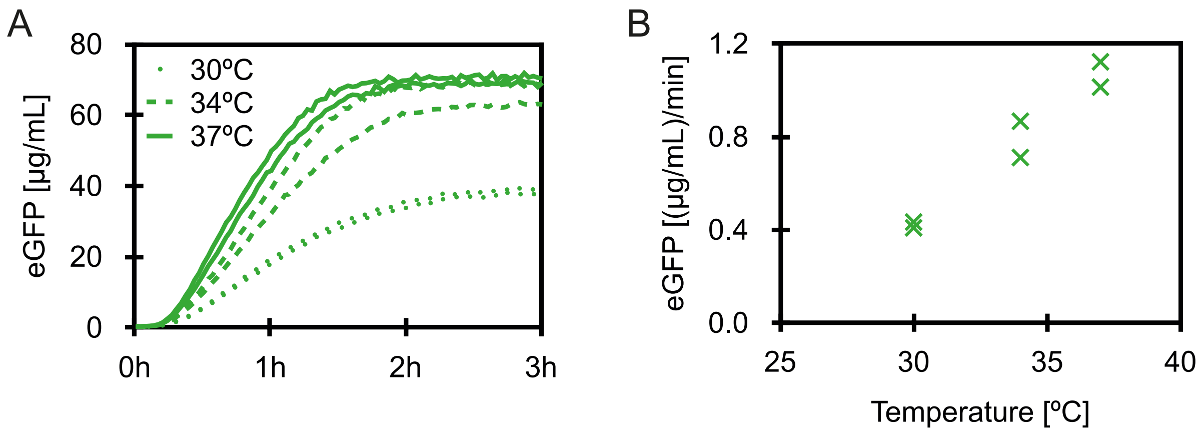

Supplementary Figure 2: **Comparison of eGFP expression at different temperatures in batch reactions:**

(A) eGFP expression over time, **(B)** eGFP expression rates. Each data point represents technical replicate.

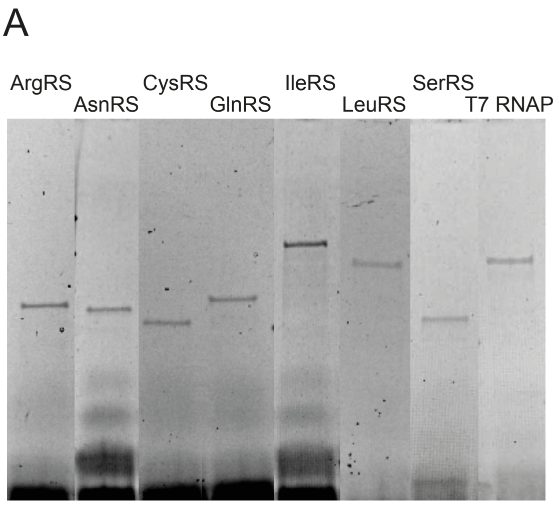

B

	Expected size [kDa]	Measured size [kDa]
ArgRS	64.7	62.5
AsnRS	52.6	55.4
CysRS	52.2	51.1
GlnRS	63.5	66.2
IleRS	104.3	111.6
LeuRS	97.2	105.4
SerRS	48.4	51.3
T7 RNAP	98.9	109.2

Supplementary Figure 3: ***In vitro* expression of different self-regenerated proteins:** **(A)** SDS-PAGE gel of *in vitro* synthesized proteins labeled with FluoroTect GreenLys. Full protein gels are provided in a Source data file. **(B)** Mass analysis of the expressed proteins. Proteins ArgRS, AsnRS, CysRS, GlnRS and LeuRS were expressed analyzed by PAGE gel twice, SerRS, IleRS and T7 RNAP were expressed analyzed by PAGE gel once with similar results.

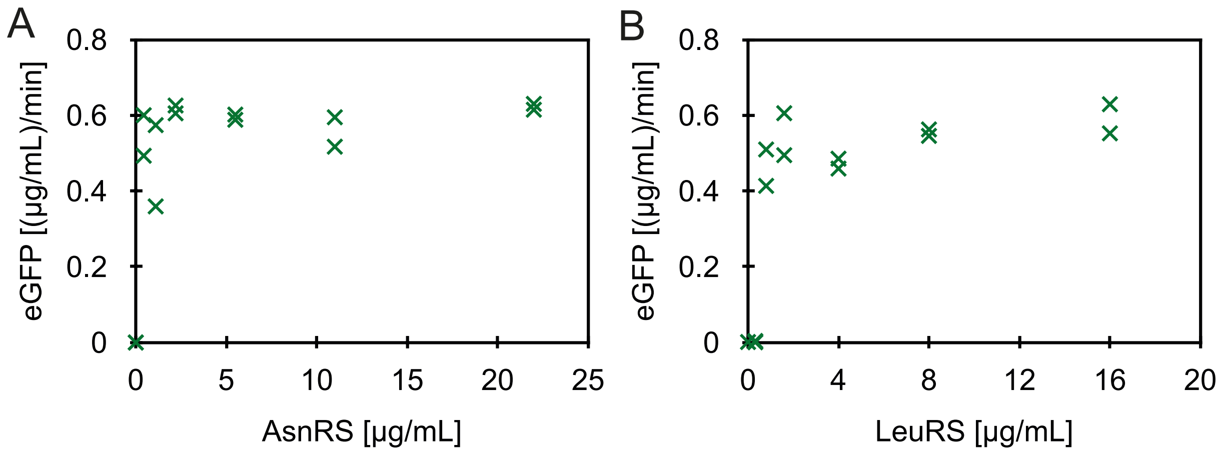

Supplementary Figure 4: **Comparison of eGFP expression rates in batch reactions at different components concentrations:**

(A) AsnRS, **(B)** LeuRS. Each data point represents a technical replicate. Source data is available in the Source Data file.

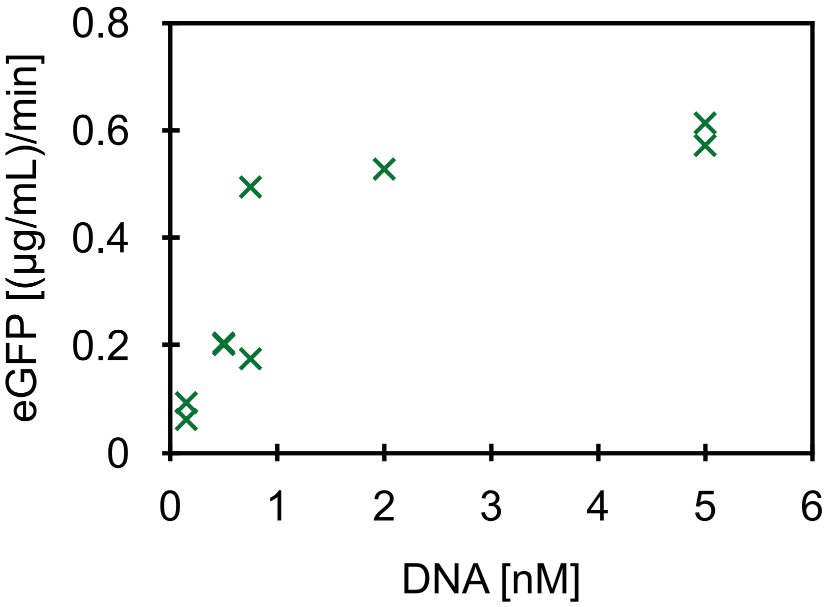

Supplementary Figure 5: Comparison of eGFP expression rates in batch reactions at different DNA template concentrations. Each data point represents a technical replicate. Source data is available in the Source Data file.

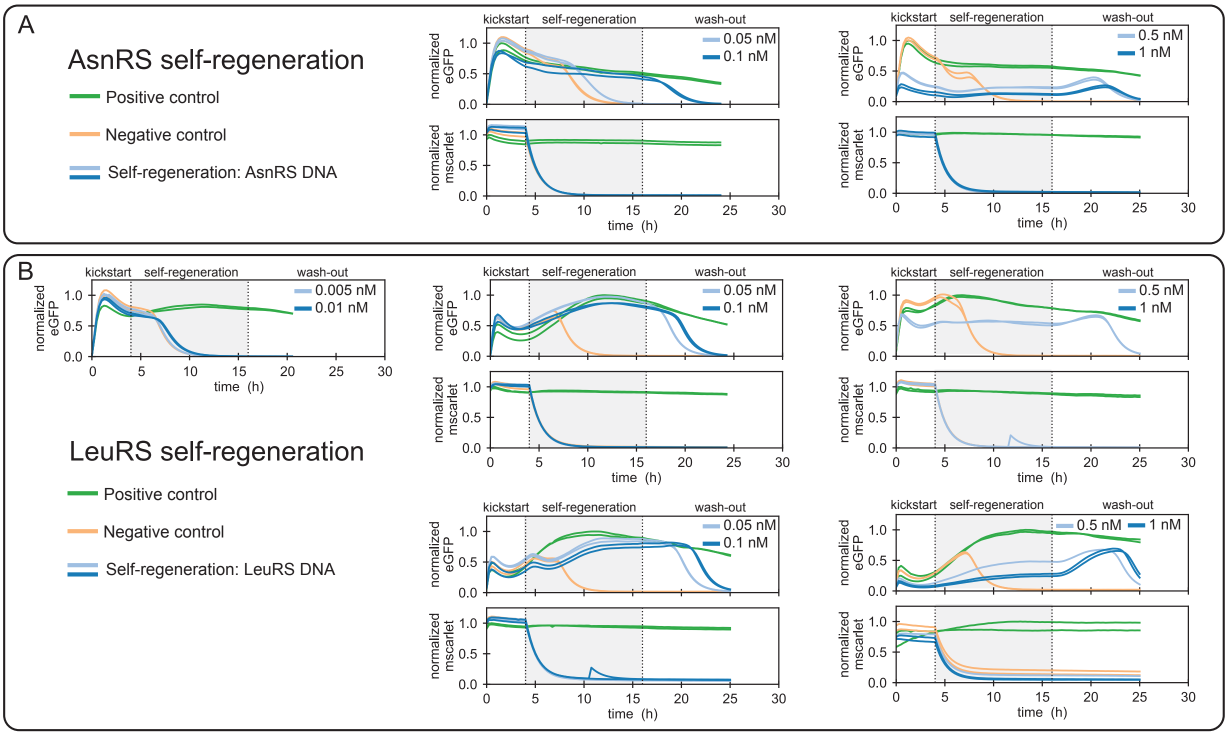

Supplementary Figure 6: **Aminoacyl-tRNA synthetase regeneration:**

Summary of all **(A)** AsnRS and **(B)** LeuRS regeneration experiments and their corresponding mScarlet traces. The level of eGFP intensity is normalised to the maximum intensity obtained in the positive control (positive control: green, negative control: yellow, self-regeneration: blue). PURE system compositions used for the different experiments are given in Supplementary Table 3. 2 nM eGFP DNA template was used, and aaRS DNA template concentrations are indicated in the corresponding graphs. Source data is available in the Source Data file.

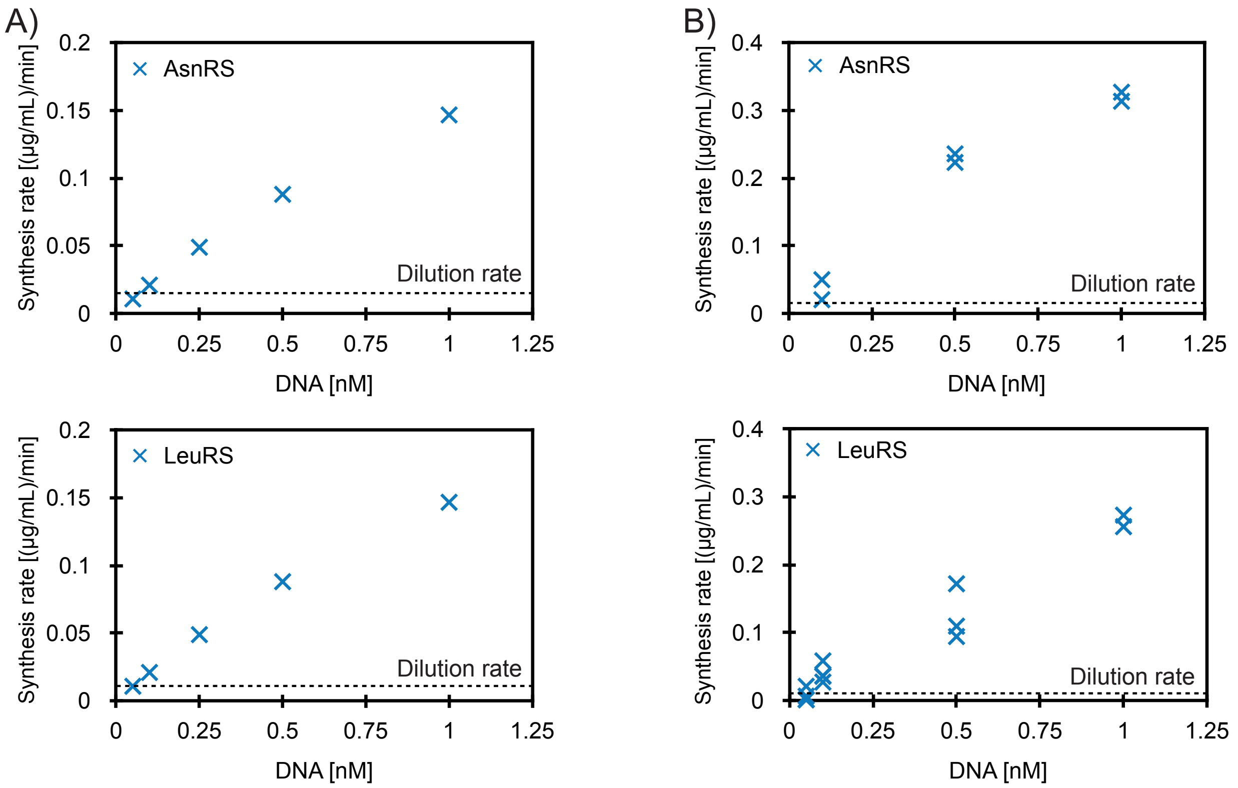

Supplementary Figure 7: **Synthesis rate for single component expression:**

(A) Theoretical synthesis rate for single components expression, calculated based on eGFP synthesis rate in a microfluidic chemostat (0.44 (μg/mL)/min) and DNA loading in DNA saturated system. **(B)** Estimated synthesis for AsnRS and LeuRS at different DNA concentrations based on the difference in eGFP synthesis rate for positive control and self-regeneration experiment at 15 hours. The eGFP synthesis rate was calculated based on an eGFP calibration curve (Supplementary Fig. 24B) and dilution rate. Dashed line represents the dilution rate of the given components based on the input component concentration (Supplementary Table 4).

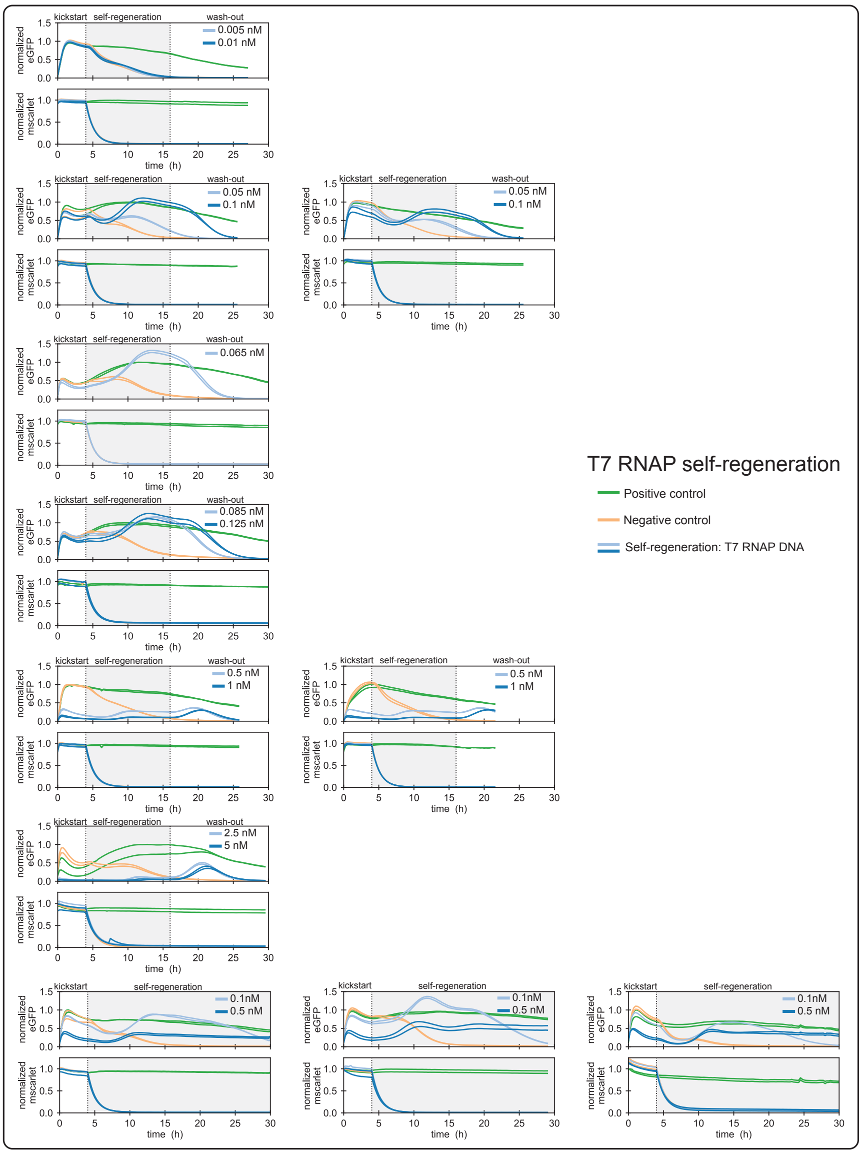

Supplementary Figure 8: **T7 RNAP regeneration:**

Results of regeneration experiments for all T7 RNAP DNA concentrations shown in Figure 3E, together with their corresponding mScarlet traces. The level of eGFP intensity is normalised to the maximum intensity obtained in the positive control (positive control: green, negative control: yellow, self-regeneration: blue). PURE system compositions used for different experiments are given in Supplementary Table 3. 2 nM of eGFP DNA template was used, and T7 RNAP DNA template concentrations are indicated in the corresponding graphs. Source data is available in the Source Data file.

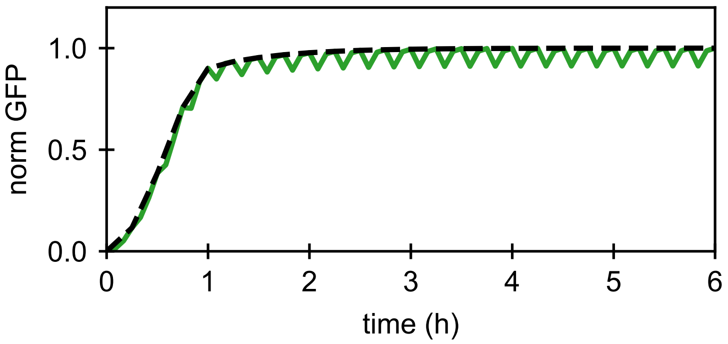

Supplementary Figure 9: **Chemostat simulations:**

The chemostat is simulated by periodically diluting and replenishing species, and solving ODEs between the dilution steps. This leads to a sawtooth curve (green). Experimental measurements are taken immediately before each dilution step, which results in a smooth observation (dashed black line).

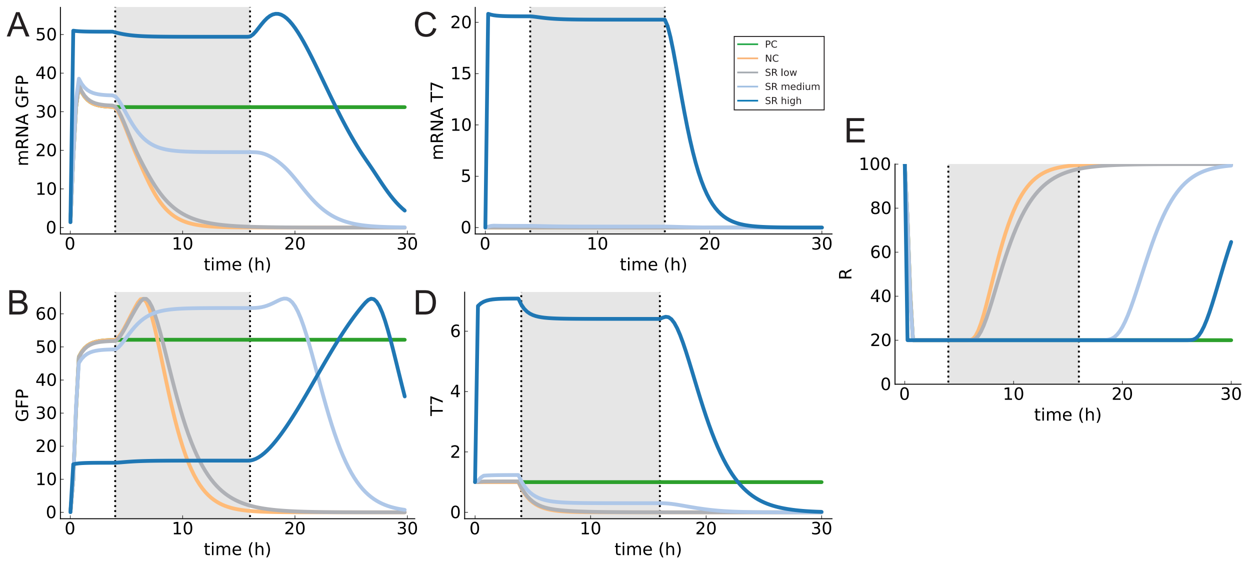

Supplementary Figure 10: **Simulation results for mRNA, protein and resource concentration:**

(A,B) Simulation results showing eGFP and **(C,D)** T7 RNAP mRNA and protein concentrations, as well as concentration of resource R **(E)**. Parameter values were $\alpha = 0.7$, $\beta = 0.07$, $K = 1$ and initial conditions $R_0 = 100$, $p_T = 1$, $d_G = 2$, with all other species set to zero. The three concentrations of d_T are 0.001, 0.01, and 1, corresponding to the labels 'low', 'medium', and 'high', respectively.

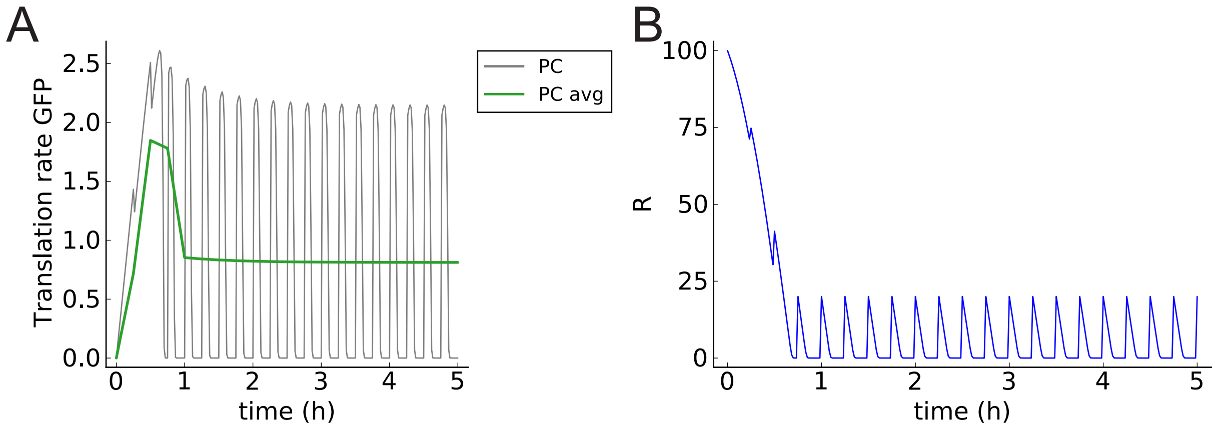

Supplementary Figure 11: **Derivatives can be directly calculated in the model, yielding rates of transcription and translation:**

(A) Periodic dilution of the chemostat leads to variations in rates, so we report the rates averaged over every period. **(B)** Translation of GFP occurs in a resource-limited regime, as resources are fully depleted over the course of each period.

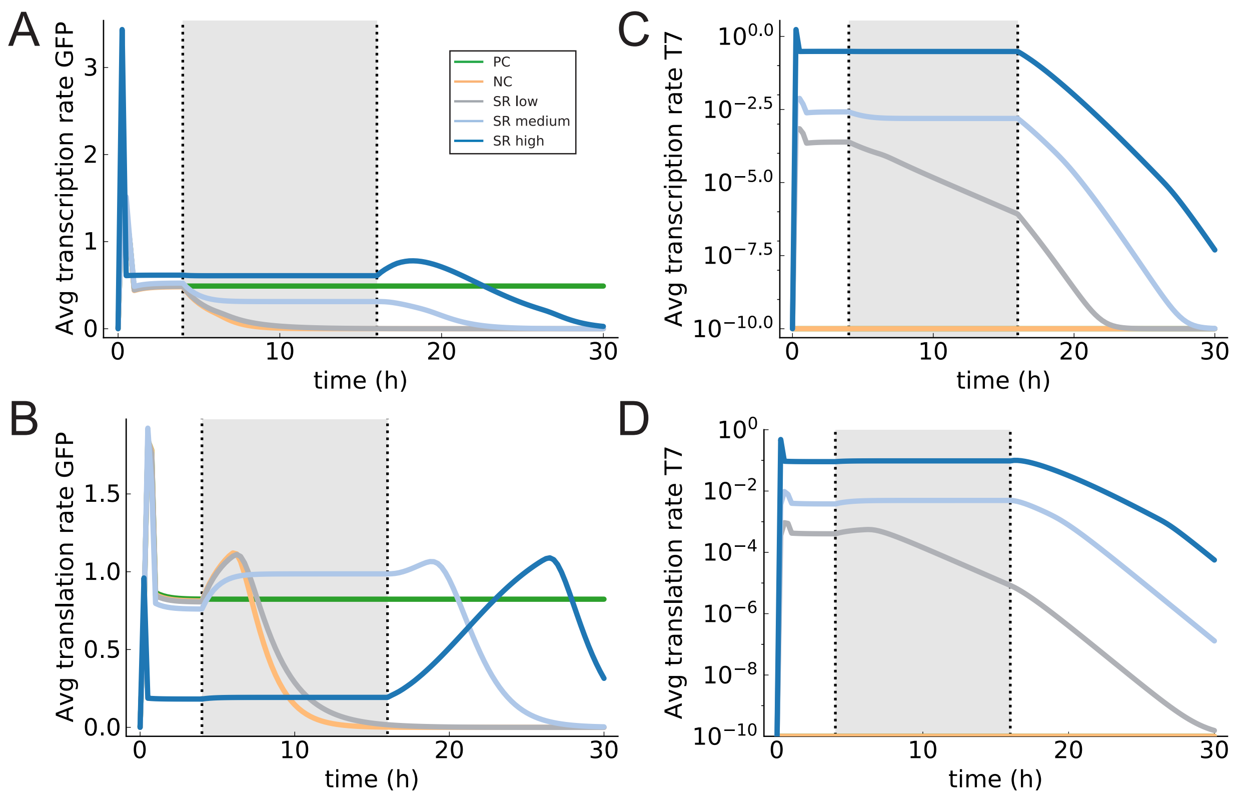

Supplementary Figure 12: **Parameter variations for the resource-dependent model:** **(A,B)** Averaged transcription and translation rates for GFP and **(C,D)** T7 RNAP, for the same parameters as in Figure 10. To make the T7 rates more clear we plotted them on a log scale, with all values smaller than 10^{-10} set to 10^{-10}.

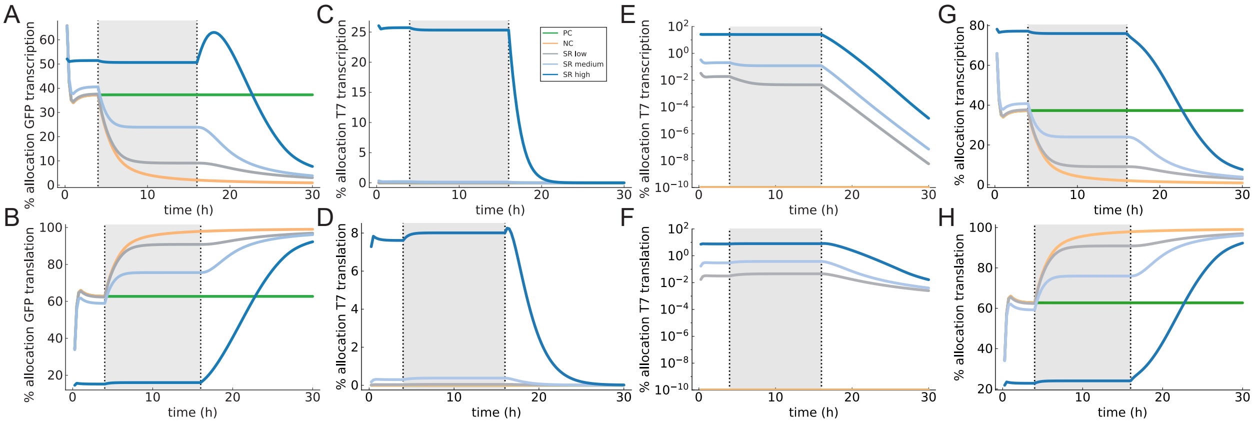

Supplementary Figure 13: **Varying resource allocation over the course of the simulation: (A,B)** We observe reallocation of resources from transcription to translation at the beginning of the self-regeneration phase. **(C,D)** Resources consumed by T7 transcription and translation are shown on linear and **(E,F)** log scales for clarity. **(G,H)** The division of resources between total transcription and total translation. As T7 RNAP is washed out after 16 hours, resource allocation tends to 100% translation.

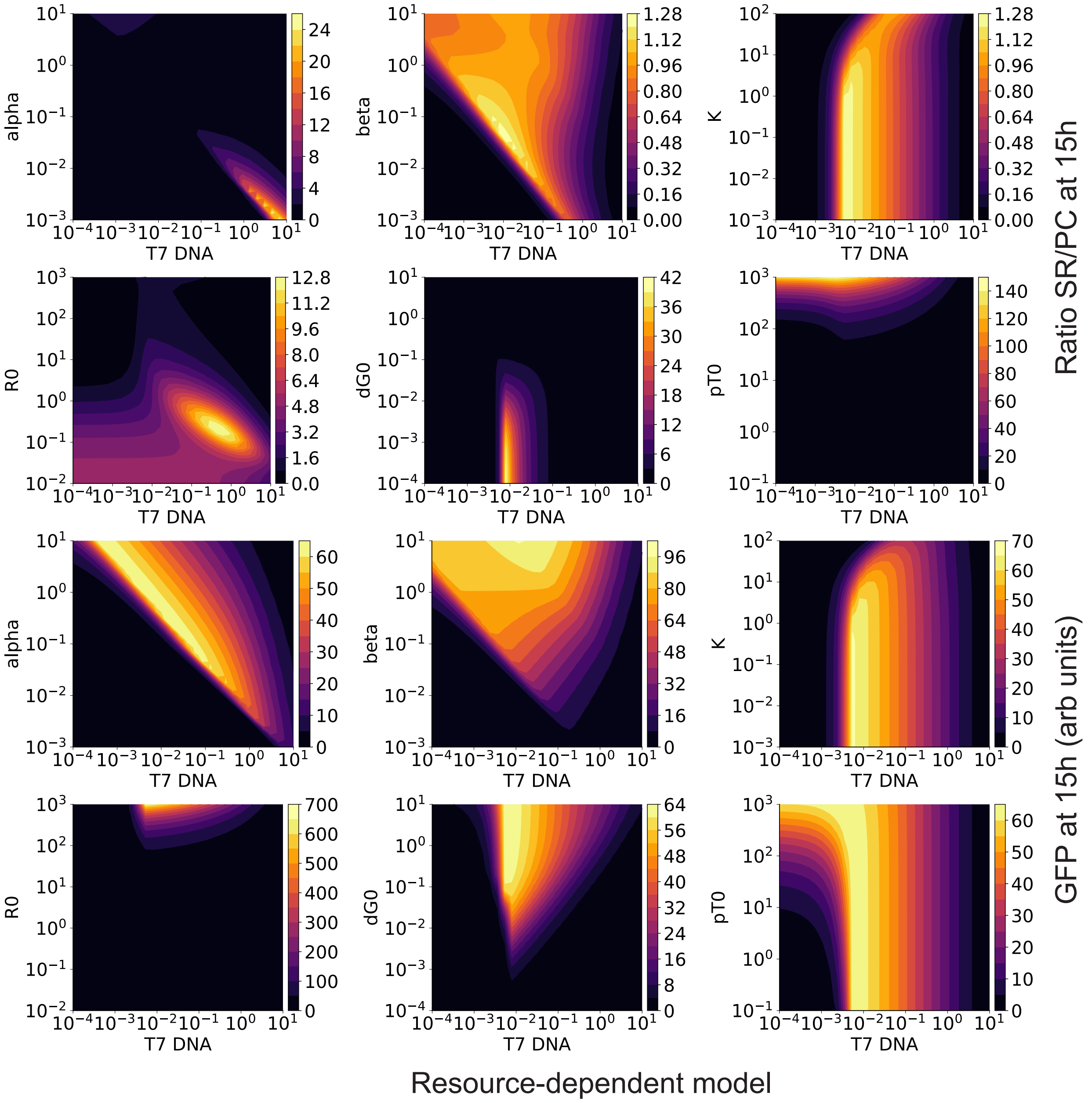

Supplementary Figure 14: **Parameter variations for the resource-dependent model:** The effects of parameter variations on SR/PC ratio (top) and total GFP yield (bottom) for the resource-dependent model. We observe that SR/PC ratios are high for small values of R_0, or for very high T7 DNA concentrations combined with low transcription rates; both these cases correspond to a resource-constrained situation.

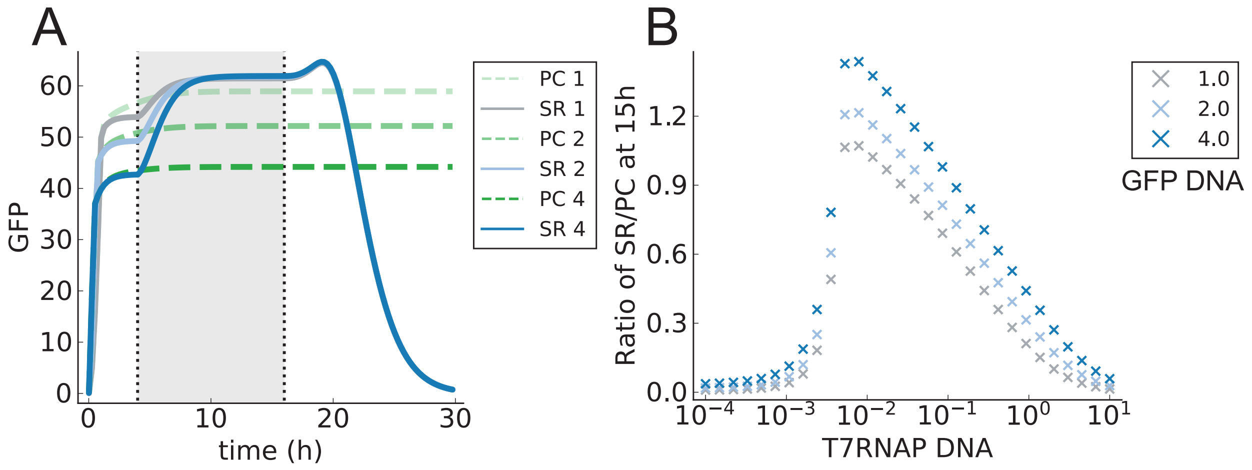

Supplementary Figure 15: **The effect of varying eGFP DNA (for values of 1, 2, and 4 nondimensional units) on the SR/PC ratio:**

(A) The resource-dependent model predicts that increasing eGFP DNA concentration lowers the positive control, as the reaction reaches steady state sooner due to faster consumption of resources.

(B) This results in an increased SR/PC ratio during the self-regeneration phase.

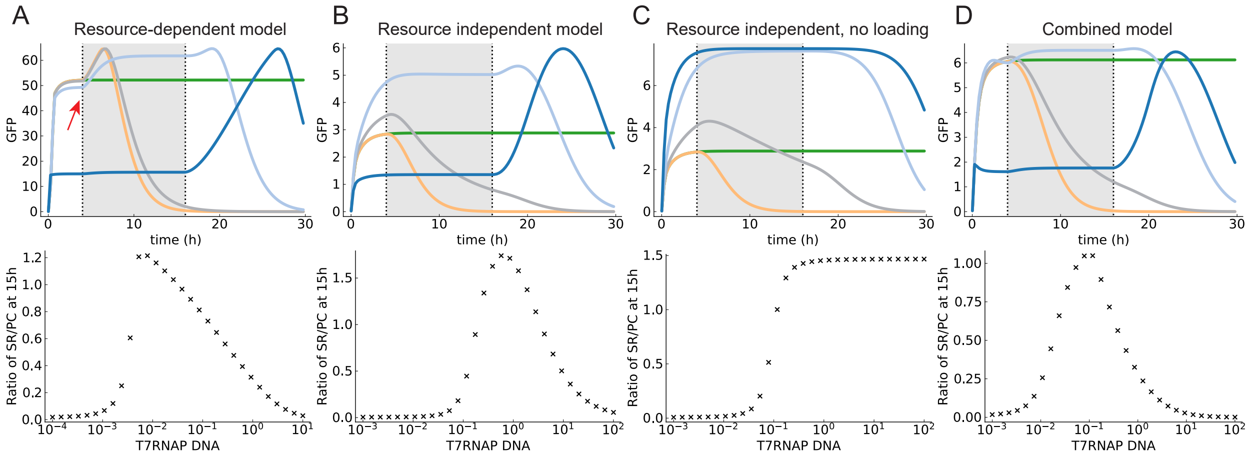

Supplementary Figure 16: **Comparison of different models:**

A resource-independent model **(B)** can also capture qualitatively similar results as the resource-dependent model **(A)**, showing a peak in eGFP production over a titration of T7 RNAP DNA. The major difference between the predicted behaviours is the rise in eGFP production after the beginning of the self-regeneration phase for the resource-dependent model (indicated by the red arrow), compared with the immediate rise at the beginning of the kick-start for the resource-independent model. Both models rely on coupling of eGFP and T7 RNAP production, through either a shared resource or enzyme. Removing the coupling eliminates the experimentally-observed optimum **(C)**. In reality both effects are likely to be present, and a combined model **(D)** can also capture experimental results, at the expense of increased complexity.

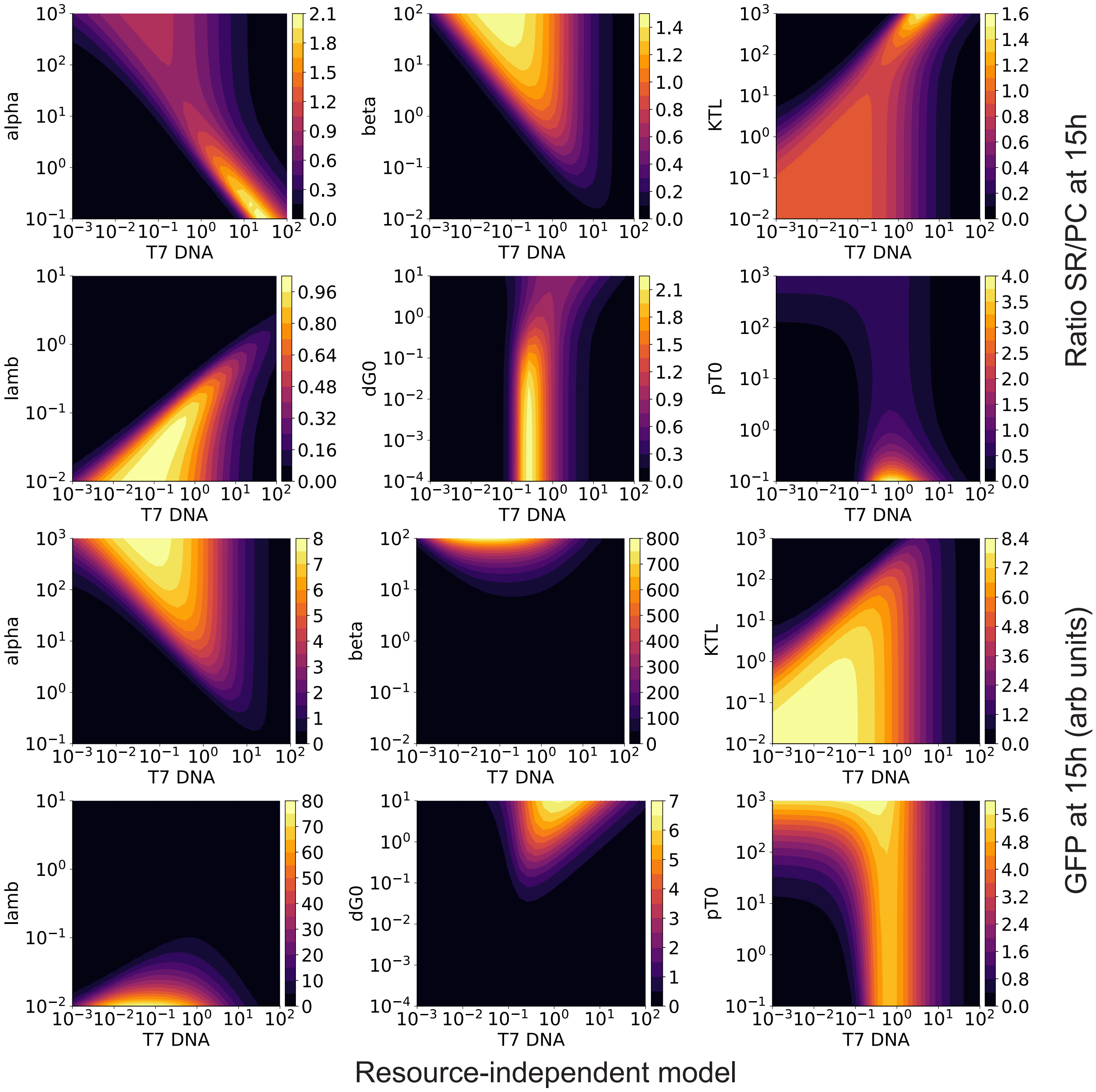

Supplementary Figure 17: **Parameter variations for the resource-independent model:** The effects of parameter variations on SR/PC ratio (top) and total GFP yield (bottom) for the resource-independent model. The variations are broadly similar to the resource-dependent model for the shared parameters α, β, dG_0, and pT_0. The behaviour of λ, the activity decay constant, is opposite to that of R_0 for the single resource model, as both parameters qualitatively limit the reaction lifetime. Finally, the model is sensitive to variations in the translation saturation constant K_{TL}.

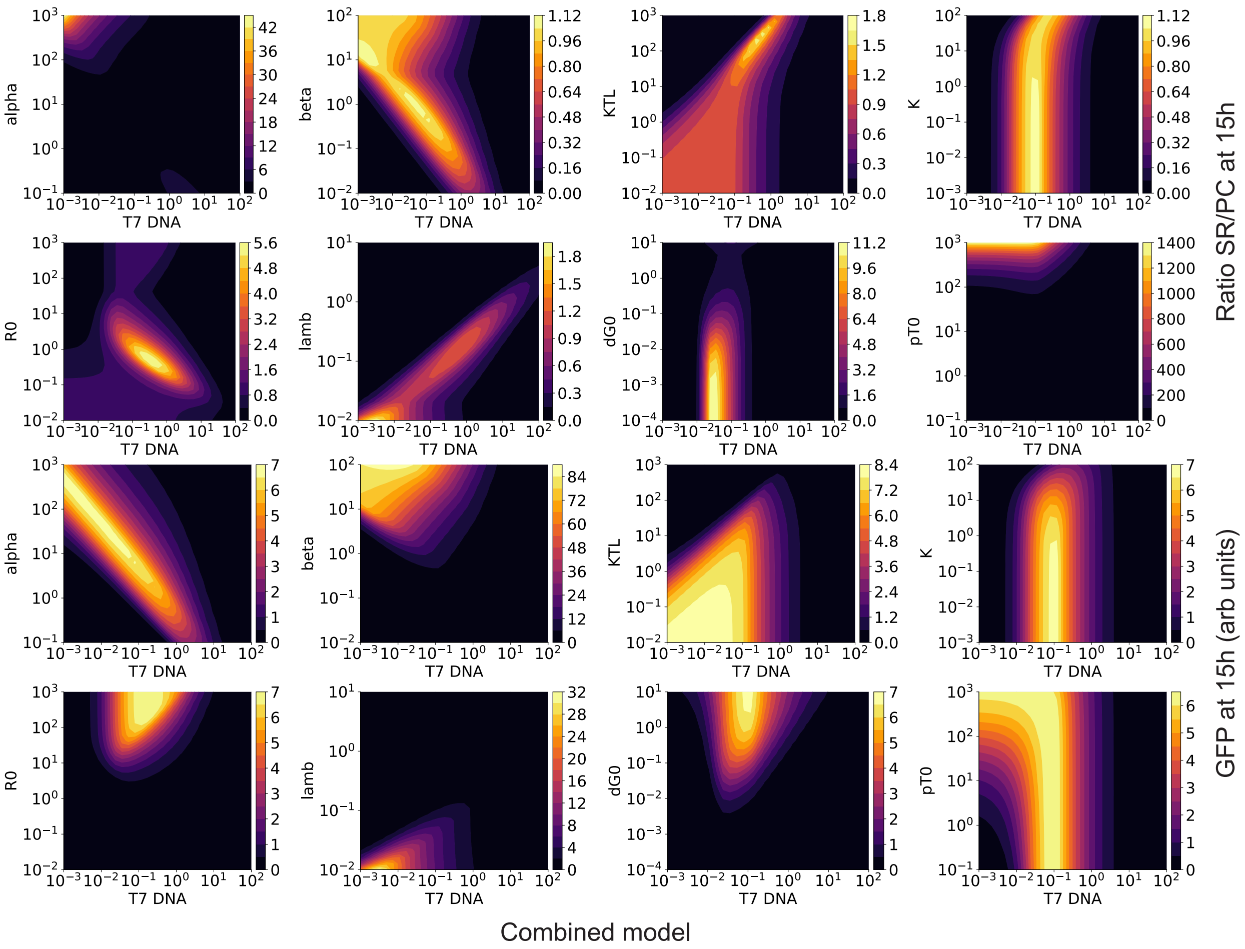

Supplementary Figure 18: **Parameter variations for the combined model:** The effects of parameter variations on SR/PC ratio (top) and total eGFP yield (bottom) for the combined model. The more complex model is sensitive to parameter variations, requiring fine-tuning to recapitulate experimental results.

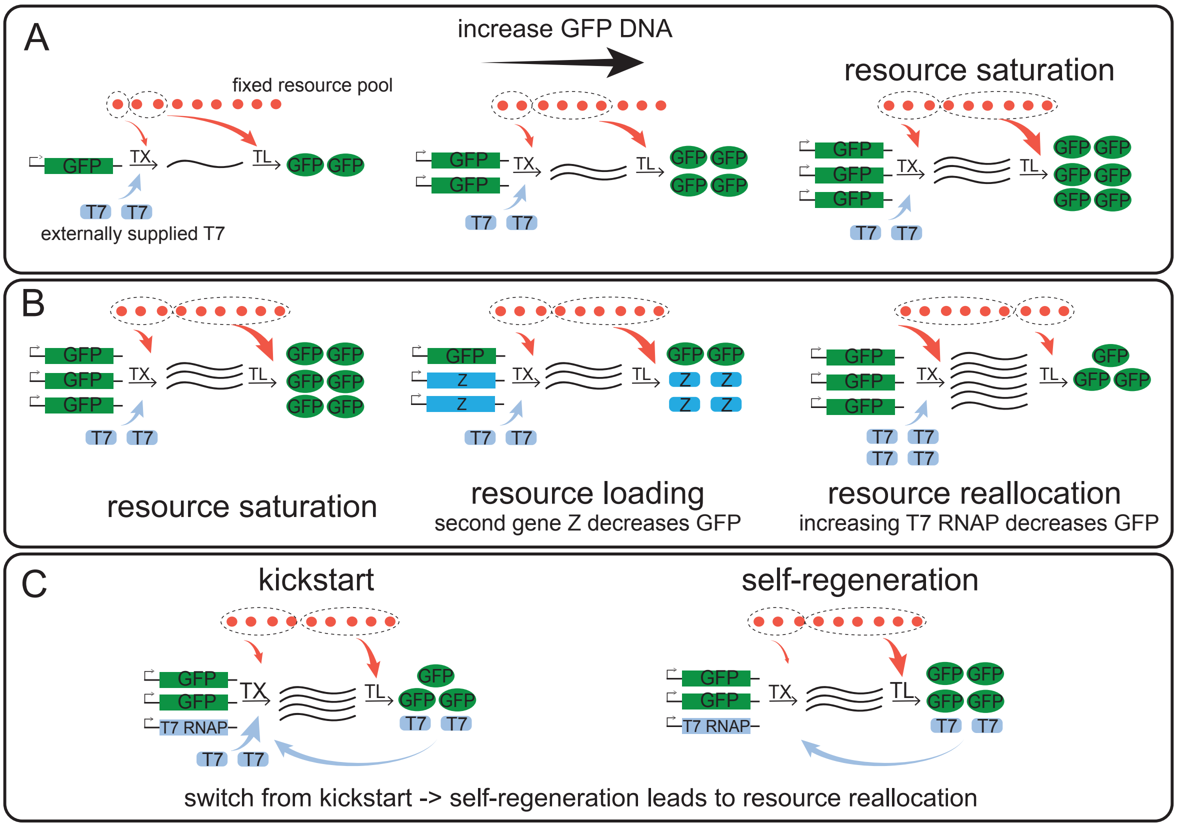

Supplementary Figure 19: **Schematic description of the concepts of resource loading and resource allocation depicted in Figure 3G:**

(A) With increasing DNA concentration, the fixed resource pool gets saturated. **(B)** Resource loading is the distribution of a limited resource between two genes. Addition of different gene Z to GFP DNA leads to a decrease in the production of GFP. Resource allocation is the distribution of a limited resource between transcription (TX) and translation (TL). Increase in T7 RNAP concentration leads to redistribution of the resources from translation to transcription, and therefore decrease in GFP production. **(C)** Difference between resource distribution in the kickstart and self-regeneration phase. In the kickstart phase, T7 RNAP is both synthesised and supplied in the PURE system pushing the resources to transcription. In the self-regeneration phase, no T7 RNAP is provided in the ΔPURE leading to release of resources from transcription and an increase in GFP production.

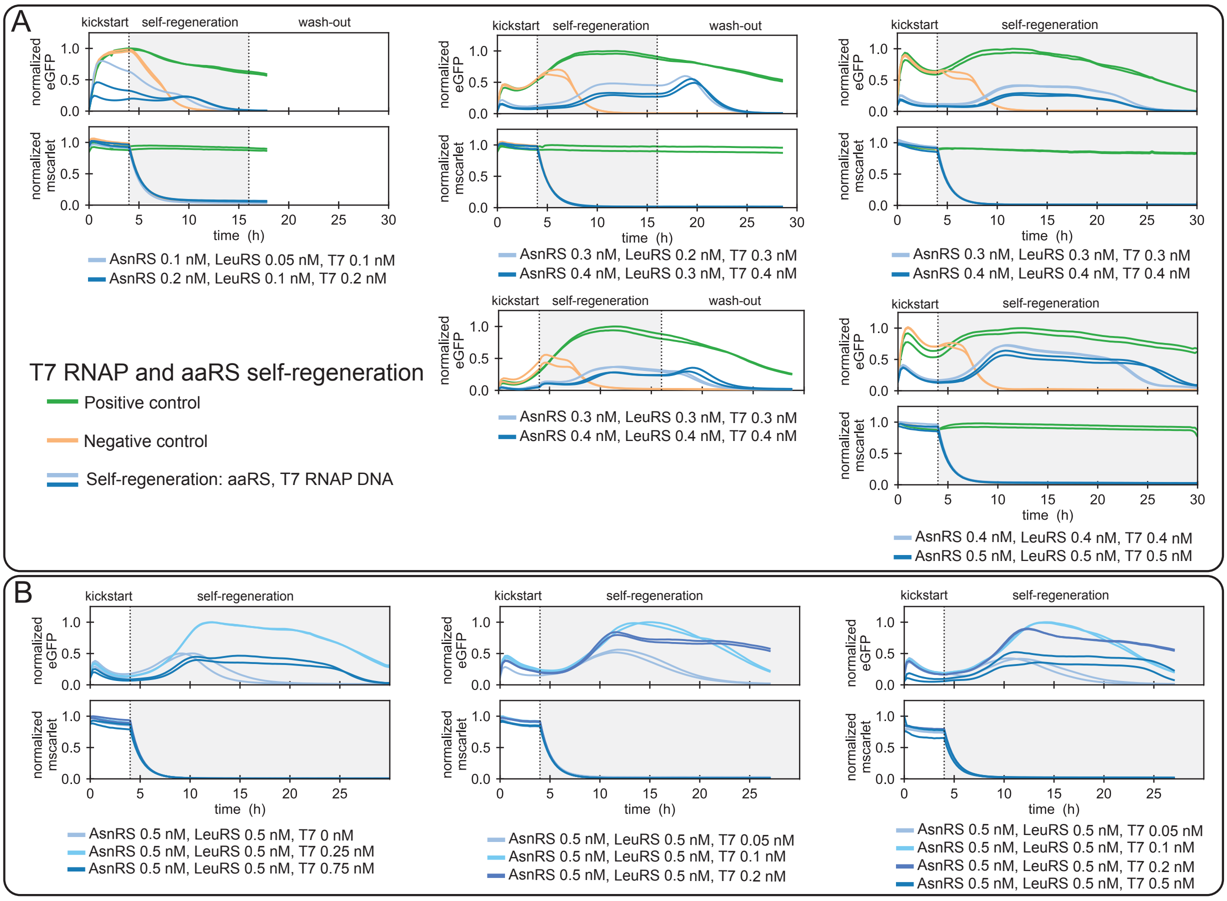

Supplementary Figure 20: **Multi-component regeneration:**

Result summary of regeneration experiments with multiple components being regenerated. Regeneration of AsnRS, LeuRS and T7 RNAP is shown in **(A)**. Titration of T7 RNAP DNA template is depicted in **(B)**. The corresponding mScarlet traces for the given experiments are shown. The level of eGFP intensity is normalised to the maximum intensity obtained in the positive control or to the overall maximum intensity if no positive control was included (positive control: green, negative control: yellow, self-regeneration: blue). PURE composition used for the regeneration experiments are given in Supplementary Table 3. 2 nM of eGFP DNA template was used, and other DNA template concentrations are indicated in the corresponding graphs. Source data is available in the Source Data file.

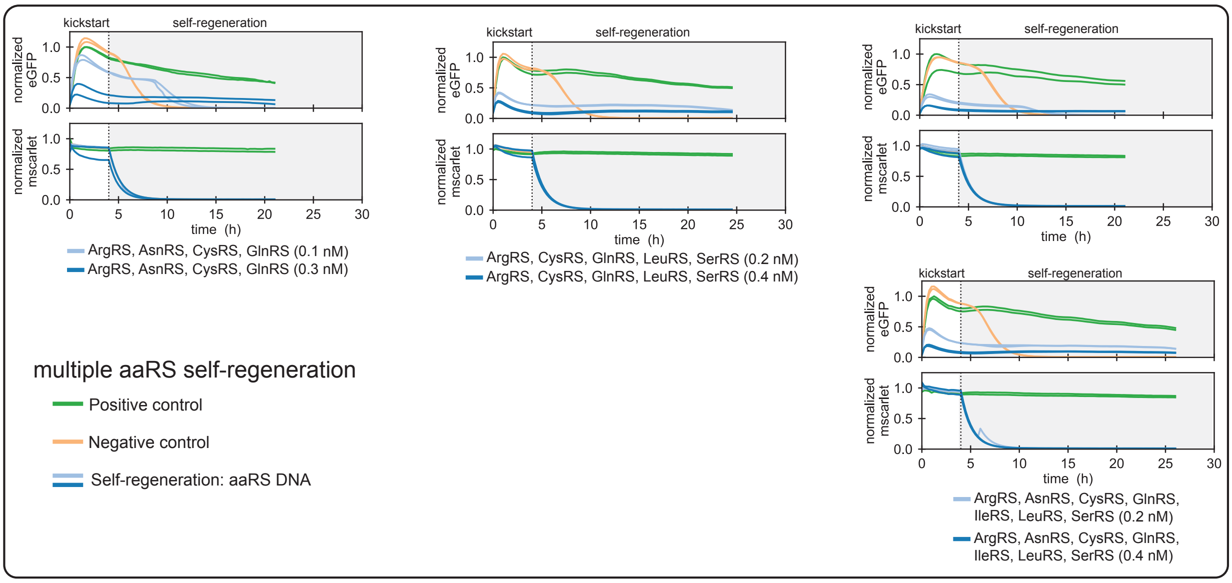

Supplementary Figure 21: **Multiple aaRSs protein regeneration:**
Result summary of multiple aaRSs protein regeneration experiments. The corresponding mScarlet traces for the given experiments are shown. The level of eGFP intensity is normalised to the maximum intensity obtained in the positive control (positive control: green, negative control: yellow, self-regeneration: blue). PURE composition used for the regeneration experiments are given in Supplementary Table 3. 2 nM of eGFP DNA template was used, and other DNA template concentrations are indicated in the corresponding graphs. Source data is available in the Source Data file.

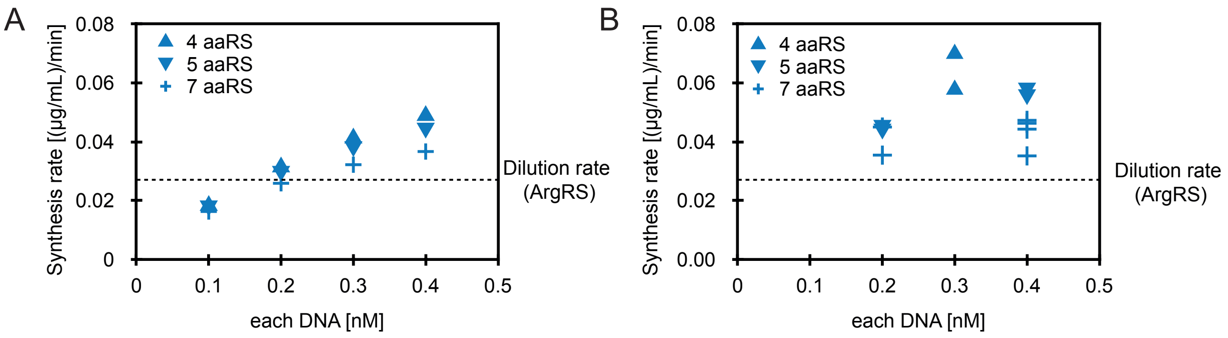

Supplementary Figure 22: **Synthesis rate for single components in multi-component expression:**

(A) Theoretical synthesis rate for single component in multiple components expression, calculated based on eGFP synthesis rate in a microfluidic chemostat (0.44 (μg/mL)/min) and DNA loading in DNA saturated system. **(B)** Estimated synthesis for each component at different DNA concentrations based on the difference in eGFP synthesis rate for positive control and self-regeneration experiment at 15 hours. The eGFP synthesis rate was calculated based on an eGFP calibration curve (Supplementary Fig. 24B) and dilution rate. Dashed line represents the dilution rate of the highest concentrated component (ArgRS), based on the input component concentrations (Supplementary Table 4).

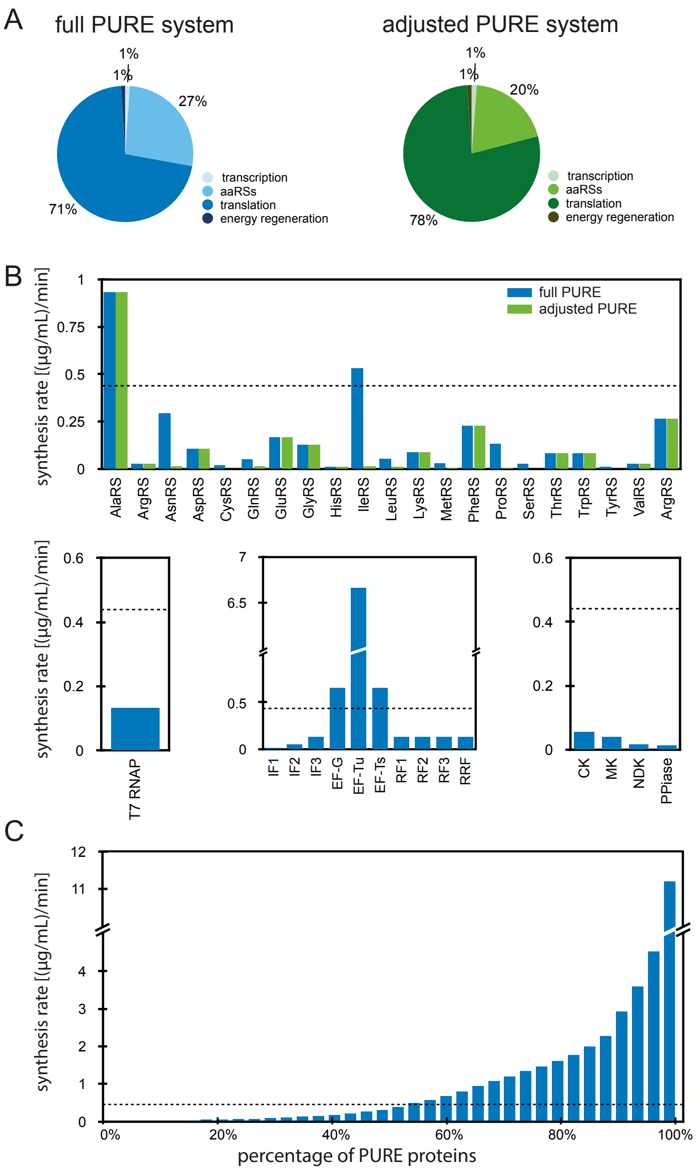

Supplementary Figure 23: **PURE system composition and synthesis rates:** **(A)** Schematic representation of the composition of the full PURE system (blue) and adjusted PURE system (green) used for multiple components regeneration. Detailed compositions are given in Supplementary Table 3. **(B)** Estimated minimal required synthesis rate of each PURE component based on dilution rate of each component (Supplementary Table 4) in comparison to the PURE synthesis rate (dashed line). **(C)** Estimated required cumulative synthesis rate for the regeneration of different PURE protein percentage in comparison to the PURE synthesis rate (dashed line). The PURE synthesis rate was calculated based on eGFP expression in a microfluidic chemostat.

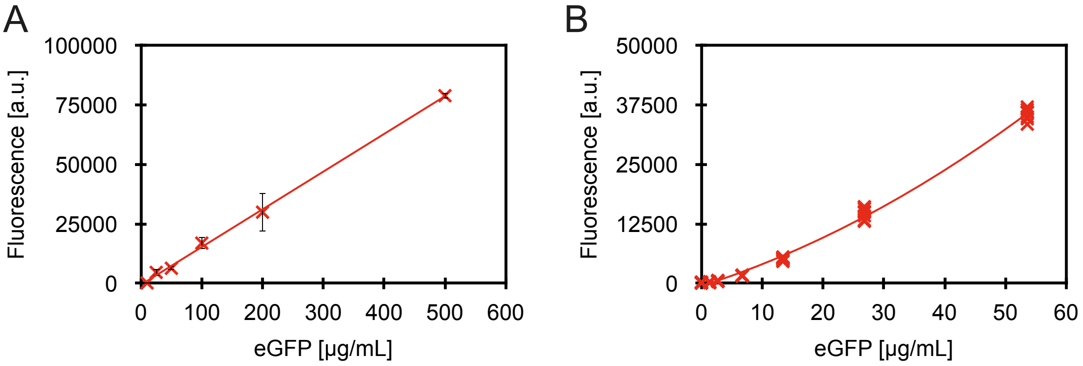

Supplementary Figure 24: **Calibration curve for different eGFP (TP790050, AMS Biotechnology) concentrations in PBS**:

(A) Plate-reader: the standard curve was produced by measuring fluorescence over 60 min with the same settings as for *in vitro* expression. Excitation and emission wavelengths were 488 nm and 507 nm, respectively. Experiments were performed in triplicates. Fluorescence measurements in the first 20 min were not considered. Values are mean ± s.d. (n = 3 technical replicates). **(B)** Microfluidic device: the standard curve was produced by measuring fluorescence over 10 min with the same settings as for *in vitro* expression. Each point represents individual reactor. The fit errors were not propagated as they were negligible compared to experimental errors. Source data is available in the Source Data file.

Supplementary Table 1: Microfluidic chip operations for self-regeneration experiments, including positive, and negative controls

Initial fill

Step	Operation	Solution	Ring number
	Repeat the following steps every 15 min		
0B	Energy solution addition		
	Flush rings	Buffer	1-8
	Flush rings	Energy solution	1-8
0C	PURE solution addition		
	Load 40% (Flush through outlet 60%)	PURE	1-8
0D	DNA solution addition		
	Load 20% (Flush through outlet 20%)	GFP DNA	1-4
	Load 20% (Flush through outlet 20%)	GFP DNA & protein DNA 1	5-6
	Load 20% (Flush through outlet 20%)	GFP DNA & protein DNA 2	7-8
0E	Mix		
	Follow with dilution steps after 15 min		

kickstart

Step	Operation	Solution	Ring number
	Repeat the following steps every 15 min		
1A	Image each reactor		
	Replace 20% of the ring content		
1B	Energy solution addition		
	Flush through outlet 20%	Buffer	1-8
	Load 8% (Flush through outlet 20%)	Energy solution	1-8
1C	PURE solution addition		
	Flush through outlet 12%	Buffer	1-8
	Load 8% (Flush through outlet 12%)	PURE	1-8
1D	DNA solution addition		
	Flush through outlet 4%	Buffer	1-8
	Load 4% (Flush through outlet 4%)	GFP DNA	1-4
	Load 4% (Flush through outlet 4%)	GFP DNA & protein DNA 1	5-6
	Load 4% (Flush through outlet 4%)	GFP DNA & protein DNA 2	7-8
1E	Mix		
	Repeat from the step 1A-E		

self-regeneration

Step	Operation	Solution	Ring number
	Repeat the following steps every 15 min		
2A	Image each reactor		
	Replace 20% of the ring content		
2B	Energy solution addition		
	Flush through outlet 20%	Buffer	1-8
	Load 8% (Flush through outlet 20%)	Energy solution	1-8
2C	PURE solution addition		
	Flush through outlet 12%	Buffer	1-8
	Load 8% (Flush through outlet 12%)	PURE	1-2
	Load 8% (Flush through outlet 12%)	ΔPURE	3-8
2D	DNA solution addition		
	Flush through outlet 4%	Buffer	1-8
	Load 4% (Flush through outlet 4%)	GFP DNA	1-4
	Load 4% (Flush through outlet 4%)	GFP DNA & protein DNA 1	5-6
	Load 4% (Flush through outlet 4%)	GFP DNA & protein DNA 2	7-8
2E	Mix		
	Repeat from the step 2A-E		

wash-out

Step	Operation	Solution	Ring number
	Repeat the following steps every 15 min		
3A	Image each reactor		
	Replace 20% of the ring content		
3B	Energy solution addition		
	Flush through outlet 20%	Buffer	1-8
	Load 8% (Flush through outlet 20%)	Energy solution	1-8
3C	PURE solution addition		
	Flush through outlet 12%	Buffer	1-8
	Load 8% (Flush through outlet 12%)	PURE	1-2
	Load 8% (Flush through outlet 12%)	ΔPURE	3-8
3D	DNA solution addition		
	Flush through outlet 4%	Buffer	1-8
	Load 4% (Flush through outlet 4%)	GFP DNA	1-8
3E	Mix		
	Repeat from the step 3A-E		

Supplementary Table 2: Microfluidic chip operations with four self-regeneration experiments

Initial fill			
Step	Operation	Solution	Ring number
Repeat the following steps every 15 min			
0B	Energy solution addition		
	Flush rings	Buffer	1-8
	Flush rings	Energy solution	1-8
0C	PURE solution addition		
	Load 40% (Flush through outlet 60%)	PURE	1-8
0D	DNA solution addition		
	Load 4% (Flush through outlet 4%)	GFP DNA & protein DNA 1	1-2
	Load 4% (Flush through outlet 4%)	GFP DNA & protein DNA 2	3-4
	Load 4% (Flush through outlet 4%)	GFP DNA & protein DNA 3	5-6
	Load 4% (Flush through outlet 4%)	GFP DNA & protein DNA 4	7-8
0E	Mix		
Follow with dilution steps after 15 min			

kickstart			
Step	Operation	Solution	Ring number
Repeat the following steps every 15 min			
1A	Image each reactor		
	Replace 20% of the ring content		
1B	Energy solution addition		
	Flush through outlet 20%	Buffer	1-8
	Load 8% (Flush through outlet 20%)	Energy solution	1-8
1C	PURE solution addition		
	Flush through outlet 12%	Buffer	1-8
	Load 8% (Flush through outlet 12%)	PURE	1-8
1D	DNA solution addition		
	Flush through outlet 4%	Buffer	1-8
	Load 4% (Flush through outlet 4%)	GFP DNA & protein DNA 1	1-2
	Load 4% (Flush through outlet 4%)	GFP DNA & protein DNA 2	3-4
	Load 4% (Flush through outlet 4%)	GFP DNA & protein DNA 3	5-6
	Load 4% (Flush through outlet 4%)	GFP DNA & protein DNA 4	7-8
1E	Mix		
Repeat from the step 1A-E			

self-regeneration			
Step	Operation	Solution	Ring number
Repeat the following steps every 15 min			
2A	Image each reactor		
	Replace 20% of the ring content		
2B	Energy solution addition		
	Flush through outlet 20%	Buffer	1-8
	Load 8% (Flush through outlet 20%)	Energy solution	1-8
2C	PURE solution addition		
	Flush through outlet 12%	Buffer	1-8
	Load 8% (Flush through outlet 12%)	ΔPURE	1-2
2D	DNA solution addition		
	Flush through outlet 4%	Buffer	1-8
	Load 4% (Flush through outlet 4%)	GFP DNA & protein DNA 1	1-2
	Load 4% (Flush through outlet 4%)	GFP DNA & protein DNA 2	3-4
	Load 4% (Flush through outlet 4%)	GFP DNA & protein DNA 3	5-6
	Load 4% (Flush through outlet 4%)	GFP DNA & protein DNA 4	7-8
2E	Mix		
Repeat from the step 2A-E			

Supplementary Table 3: PURE system formulations used

SR experiment		PURE system	T7 RNAP	AsnRS	LeuRS	aaRSs + T7 RNAP	aaRSs
Protein			Concentration [µg/ml]				
AlaRS	Alanyl-tRNA synthetase	70.0	70.0	70.0	70.0	70.0	70.0
ArgRS	Arginyl-tRNA synthetase	2.0	2.0	2.0	2.0	2.0	2.0
AsnRS	Asparaginyl-tRNA synthetase	22.0	22.0	1.1	22.0	1.1	1.1
AspRS	Aspartate-tRNA synthetase	8.0	8.0	8.0	8.0	8.0	8.0
CysRS	Cysteinyl-tRNA synthetase	1.2	1.2	1.2	1.2	1.2	0.1
GlnRS	Glutaminyl-tRNA synthetase	3.8	3.8	3.8	3.8	3.8	1.0
GluRS	Glutamyl-tRNA synthetase	12.6	12.6	12.6	12.6	12.6	12.6
GlyRS	Glycyl-tRNA synthetase	9.6	9.6	9.6	9.6	9.6	9.6
HisRS	Histidyl-tRNA synthetase	0.8	0.8	0.8	0.8	0.8	0.8
IleRS	Isoleucyl-tRNA synthetase	40.0	40.0	40.0	40.0	40.0	1.0
LeuRS	Leucyl-tRNA synthetase	4.0	4.0	4.0	0.8	0.8	0.8
LysRS	Lysyl-tRNA synthetase	6.4	6.4	6.4	6.4	6.4	6.4
MetRS	Methionine--tRNA ligase	2.3	2.3	2.3	2.3	2.3	0.4
PheRS	Phenylalanyl-tRNA synthetase	17.0	17.0	17.0	17.0	17.0	17.0
ProRS	Prolyl-tRNA synthetase	10.0	10.0	10.0	10.0	10.0	0.4
SerRS	Seryl-tRNA synthetase	1.9	1.9	1.9	1.9	1.9	0.2
ThrRS	Threonyl-tRNA synthetase	6.2	6.2	6.2	6.2	6.2	6.2
TrpRS	Tryptophanyl-tRNA synthetase	6.3	6.3	6.3	6.3	6.3	6.3
TyrRS	Tyrosyl-tRNA synthetase	0.6	0.6	0.6	0.6	0.6	0.1
ValRS	Valyl-tRNA synthetase	1.8	1.8	1.8	1.8	1.8	1.8
IF1	Initiation factor 1	1.0	1.0	1.0	1.0	1.0	1.0
IF2	Initiation factor 2	4.0	4.0	4.0	4.0	4.0	4.0
IF3	Initiation factor 3	10.0	10.0	10.0	10.0	10.0	10.0
EF-G	Elongation factor G	50.0	50.0	50.0	50.0	50.0	50.0
EF-Tu	Elongation factor Tu	500.0	500.0	500.0	500.0	500.0	500.0
EF-Ts	Elongation factor Ts	50.0	50.0	50.0	50.0	50.0	50.0
RF1	Release factor 1	10.0	10.0	10.0	10.0	10.0	10.0
RF2	Release factor 2	10.0	10.0	10.0	10.0	10.0	10.0
RF3	Release factor 3	10.0	10.0	10.0	10.0	10.0	10.0
RRF	Ribosome recycling factor	10.0	10.0	10.0	10.0	10.0	10.0
MTF	Methionyl-tRNA formyltransferase	20.0	20.0	20.0	20.0	20.0	20.0
CK	Creatine kinase	4.0	4.0	4.0	4.0	4.0	4.0
MK	Adenylate kinase (Myokinase)	3.0	3.0	3.0	3.0	3.0	3.0
NDK	Nucleotide diphosphate kinase	1.1	1.1	1.1	1.1	1.1	1.1
PPiase	Inorganic pyrophosphatase	1.0	1.0	1.0	1.0	1.0	1.0
T7 RNAP	T7 RNA polymerase	10.0	10.0	10.0	10.0	10.0	10.0

Supplementary Table 4: Calculated dilution rates based on concentrations in Table 3

SR experiment		PURE system	T7 RNAP	AsnRS	LeuRS	aaRSs + T7 RNAP	aaRSs
Protein			Dilution rate [(μg/mL)/min]				
AlaRS	Alanyl-tRNA synthetase	0.93	0.93	0.93	0.93	0.93	0.93
ArgRS	Arginyl-tRNA synthetase	0.03	0.03	0.03	0.03	0.03	0.027
AsnRS	Asparaginyl-tRNA synthetase	0.29	0.29	0.015	0.29	0.015	0.015
AspRS	Aspartate-tRNA synthetase	0.11	0.11	0.11	0.11	0.11	0.11
CysRS	Cysteinyl-tRNA synthetase	0.02	0.02	0.02	0.02	0.02	0.001
GlnRS	Glutaminyl-tRNA synthetase	0.05	0.05	0.05	0.05	0.05	0.013
GluRS	Glutamyl-tRNA synthetase	0.17	0.17	0.17	0.17	0.17	0.17
GlyRS	Glycyl-tRNA synthetase	0.13	0.13	0.13	0.13	0.13	0.13
HisRS	Histidyl-tRNA synthetase	0.01	0.01	0.01	0.01	0.01	0.01
IleRS	Isoleucyl-tRNA synthetase	0.53	0.53	0.53	0.53	0.53	0.013
LeuRS	Leucyl-tRNA synthetase	0.05	0.05	0.05	0.011	0.011	0.011
LysRS	Lysyl-tRNA synthetase	0.09	0.09	0.09	0.09	0.09	0.09
MetRS	Methionine--tRNA ligase	0.03	0.03	0.03	0.03	0.03	0.005
PheRS	Phenylalanyl-tRNA synthetase	0.23	0.23	0.23	0.23	0.23	0.23
ProRS	Prolyl-tRNA synthetase	0.13	0.13	0.13	0.13	0.13	0.005
SerRS	Seryl-tRNA synthetase	0.03	0.03	0.03	0.03	0.03	0.003
ThrRS	Threonyl-tRNA synthetase	0.08	0.08	0.08	0.08	0.08	0.08
TrpRS	Tryptophanyl-tRNA synthetase	0.08	0.08	0.08	0.08	0.08	0.08
TyrRS	Tyrosyl-tRNA synthetase	0.01	0.01	0.01	0.01	0.01	0.001
ValRS	Valyl-tRNA synthetase	0.02	0.02	0.02	0.02	0.02	0.02
IF1	Initiation factor 1	0.01	0.01	0.01	0.01	0.01	0.01
IF2	Initiation factor 2	0.05	0.05	0.05	0.05	0.05	0.05
IF3	Initiation factor 3	0.13	0.13	0.13	0.13	0.13	0.13
EF-G	Elongation factor G	0.67	0.67	0.67	0.67	0.67	0.67
EF-Tu	Elongation factor Tu	6.67	6.67	6.67	6.67	6.67	6.67
EF-Ts	Elongation factor Ts	0.67	0.67	0.67	0.67	0.67	0.67
RF1	Release factor 1	0.13	0.13	0.13	0.13	0.13	0.13
RF2	Release factor 2	0.13	0.13	0.13	0.13	0.13	0.13
RF3	Release factor 3	0.13	0.13	0.13	0.13	0.13	0.13
RRF	Ribosome recycling factor	0.13	0.13	0.13	0.13	0.13	0.13
MTF	Methionyl-tRNA formyltransferase	0.27	0.27	0.27	0.27	0.27	0.27
CK	Creatine kinase	0.05	0.05	0.05	0.05	0.05	0.05
MK	Adenylate kinase (Myokinase)	0.04	0.04	0.04	0.04	0.04	0.04
NDK	Nucleotide diphosphate kinase	0.01	0.01	0.01	0.01	0.01	0.01
PPiase	Inorganic pyrophosphatase	0.01	0.01	0.01	0.01	0.01	0.01
eGFP	T7 RNA polymerase	0.13	0.13	0.13	0.13	0.13	0.13

Supplementary Table 5: Replenishing schedule for modeling the three-stage experiment

Self-regeneration	Species replenished
Stage 1	R, d_T, d_G, p_T
Stage 2	R, d_T, d_G
Stage 3	R, d_G
**Positive control**	**Species replenished**
Stage 1	R, d_G, p_T
Stage 2	R, d_G, p_T
Stage 3	R, d_G, p_T

Supplementary Table 6: DNA sequences

	DNA sequence	Amplification Primers	
eGFP linear DNA fragment	gatcttaaggctagagtactaatacgactcactatagggagaccacaacggtttccctctagaaataattttgtttaacttaagaaggaggaaaaaaaATGTCTAAAGGTGAAGAATTATTCACTGGTGTTGTCCCAATTTTGGTTGAATTAGATGGTGATGTTAATGGTCACAAATTTTCTGTCTCCGGTGAAGGTGAAGGTGATGCTACTTACGGTAAATTGACCTTAAAATTTATTTGTACTACTGGTAAATTGCCAGTTCCATGGCCAACCTTAGTCACTACTTTAACTTATGGTGTTCAATGTTTTTCTAGATACCCAGATCATATGAAACAACATGACTTTTTCAAGTCTGCCATGCCAGAAGGTTATGTTCAAGAAAGAACTATTTTTTTCAAAGATGACGGTAACTACAAGACCAGAGCTGAAGTCAAGTTTGAAGGTGATACCTTAGTTAATAGAATCGAATTAAAAGGTATTGATTTTAAAGAAGATGGTAACATTTTAGGTCACAAATTGGAATACAACTATAACTCTCACAATGTTTACATCATGGCTGACAAACAAAAGAATGGTATCAAAGTTAACTTCAAAATTAGACACAACATTGAAGATGGTTCTGTTCAATTAGCTGACCATTATCAACAAAATACTCCAATTGGTGATGGTCCAGTCTTGTTACCAGACAACCATTACTTATCCACTCAATCTGCCTTATCCAAAGATCCAAACGAAAAGAGAGACCACATGGTCTTGTTAGAATTTGTTACTGCTGCTGGTATTACCCATGGTATGGATGAATTGTACAAATAAtaacgactcaggctgctacgcctgtgtactggaaaacaaaaccaaaacccaaaaaacaaaaaactgagcccattggtatcgtggaaggactctatcaaaaaaaaaaaaaaaaaaaaaaaaaaaaaac**tagcataaccccttggggcctctaaacgggtcttgaggggttttttg**	5'-GATCTTAAGGCTAGAGTACTAATACGACTCACTATAGGGAGACC-3'	5'-CAAAAAACCCCTCAAGACCCGTTTAGAG-3'
**Chi DNA**	TGGCCACCAGCAGTGGCCACCAGCAGTGGCCACCAGCAGTGGCCACCAGCAGTGAAGTGA		
**Blue**	**T7 promoter**		
**Red**	**RBS**		
**Green**	**Gene coding for protein**		
**Bold**	**T7 terminator**		

Supplementary Table 7: Primer sequences

	Amplification Primers		Extension primers	
ArgRS	5'CCTCTAGAAATAATTTTGTTTAACTTAA GAAGGAGGAAAAAAAAAATGAATATTCA GGCTCTTCTCTCAGAAAAAGTCC 3'	5'GTAGCAGCCTGAGTCGTTATTACATAC GCTCTACAGTCTCAATACCCAGCG 3'	5'GATCTTAAGGCTAGAGTACTAATACGA CTCACTATAGGGAGACCACAACGGTTT CCCTCTAGAAATAATTTTGTTTAAC 3'	5'**CAAAAAACCCCTCAAGACCCGTTTA GAGGCCCCAAGGGGTTATGCTA**GTTTT TTTTTTTTTTTTTTTTTTTTTTTTTTTTGTAG CAGCCTGAGTCG 3'
AsnRS	5'CCTCTAGAAATAATTTTGTTTAACTTAA GAAGGAGGAAAAAAAAAATGAGCGTTGT GCCTGTAGCCG 3'	5'GTAGCAGCCTGAGTCGTTATTAGAAG CTGGCGTTACGCGGAGTAC 3'	5'GATCTTAAGGCTAGAGTACTAATACGA CTCACTATAGGGAGACCACAACGGTTT CCCTCTAGAAATAATTTTGTTTAAC 3'	5'**CAAAAAACCCCTCAAGACCCGTTTA GAGGCCCCAAGGGGTTATGCTA**GTTTT TTTTTTTTTTTTTTTTTTTTTTTTTTTTGTAG CAGCCTGAGTCG 3'
CysRS	5'CCTCTAGAAATAATTTTGTTTAACTTAA GAAGGAGGAAAAAAAAAATGCTAAAAAT CTTCAATACTCTGACACGCC 3'	5'GTAGCAGCCTGAGTCGTTATTACTTAC GACGCCAGGTGGTCCC 3'	5'GATCTTAAGGCTAGAGTACTAATACGA CTCACTATAGGGAGACCACAACGGTTT CCCTCTAGAAATAATTTTGTTTAAC 3'	5'**CAAAAAACCCCTCAAGACCCGTTTA GAGGCCCCAAGGGGTTATGCTA**GTTTT TTTTTTTTTTTTTTTTTTTTTTTTTTTTGTAG CAGCCTGAGTCG 3'
GlnRS	5'CCTCTAGAAATAATTTTGTTTAACTTAA GAAGGAGGAAAAAAAAAATGAGTGAGG CAGAAGCCCGC 3'	5'GTAGCAGCCTGAGTCGTTATTACTCG CCTACTTTCGCCCAGGTATC 3'	5'GATCTTAAGGCTAGAGTACTAATACGA CTCACTATAGGGAGACCACAACGGTTT CCCTCTAGAAATAATTTTGTTTAAC 3'	5'**CAAAAAACCCCTCAAGACCCGTTTA GAGGCCCCAAGGGGTTATGCTA**GTTTT TTTTTTTTTTTTTTTTTTTTTTTTTTTTGTAG CAGCCTGAGTCG 3'
IleRS	5'CCTCTAGAAATAATTTTGTTTAACTTAA GAAGGAGGAAAAAAAAAATGAGTGACTA TAAATCAACCCTGAATTTGCC 3'	5'GTAGCAGCCTGAGTCGTTATTAGGCA AACTTACGTTTTTCACCGTCAC 3'	5'GATCTTAAGGCTAGAGTACTAATACGA CTCACTATAGGGAGACCACAACGGTTT CCCTCTAGAAATAATTTTGTTTAAC 3'	5'**CAAAAAACCCCTCAAGACCCGTTTA GAGGCCCCAAGGGGTTATGCTA**GTTTT TTTTTTTTTTTTTTTTTTTTTTTTTTTTGTAG CAGCCTGAGTCG 3'
LeuRS	5'CCTCTAGAAATAATTTTGTTTAACTTAA GAAGGAGGAAAAAAAAAATGCAAGAGCA ATACCGCCCG 3'	5'GTAGCAGCCTGAGTCGTTATTAGCCA ACGACCAGATTGAGGAGTTTAC 3'	5'GATCTTAAGGCTAGAGTACTAATACGA CTCACTATAGGGAGACCACAACGGTTT CCCTCTAGAAATAATTTTGTTTAAC 3'	5'**CAAAAAACCCCTCAAGACCCGTTTA GAGGCCCCAAGGGGTTATGCTA**GTTTT TTTTTTTTTTTTTTTTTTTTTTTTTTTTGTAG CAGCCTGAGTCG 3'
SerRS	5'CCTCTAGAAATAATTTTGTTTAACTTAA GAAGGAGGAAAAAAAAAATGCTCGATCC CAATCTGCTGC 3'	5'GTAGCAGCCTGAGTCGTTATTAGCCAA TATATTCCAGTCCGTTCATATACGG 3'	5'GATCTTAAGGCTAGAGTACTAATACGA CTCACTATAGGGAGACCACAACGGTTT CCCTCTAGAAATAATTTTGTTTAAC 3'	5'**CAAAAAACCCCTCAAGACCCGTTTA GAGGCCCCAAGGGGTTATGCTA**GTTTT TTTTTTTTTTTTTTTTTTTTTTTTTTTTGTAG CAGCCTGAGTCG 3'
T7 RNAP	5'CCTCTAGAAATAATTTTGTTTAACTTAA GAAGGAGGAAAAAAAAAATGAACACGAT TAACATCGCTAAGAACGACTTC 3'	5'GTAGCAGCCTGAGTCGTTATTACGCG AACGCGAAGTCCG 3'	5'GATCTTAAGGCTAGAGTACTAATACGA CTCACTATAGGGAGACCACAACGGTTT CCCTCTAGAAATAATTTTGTTTAAC 3'	5'**CAAAAAACCCCTCAAGACCCGTTTA GAGGCCCCAAGGGGTTATGCTA**GTTTT TTTTTTTTTTTTTTTTTTTTTTTTTTTTGTAG CAGCCTGAGTCG 3'

	**DNA sequence**
Linear DNA fragment	gatcttaaggctagagtac**taatacgactcactatagg**gagaccacaacggtttccctctagaaataattttgtttaacttaag**aaggag**gaaaaaaaa**ATG——protein——** taataacgactcaggctgctacaaaaaaaaaaaaaaaaaaaaaaaaaaaaac**tagcataaccccttggggcctctaaacgggtcttgaggggttttttg**
**Blue**	**T7 promoter**
**Red**	**RBS**
**Bold**	**T7 terminator**

Supplementary Table 8: Buffers and energy solution

PURE buffer

Compound	Catalog number	Company	Buffer A	Buffer B	HT buffer	Stock buffer	Note
			mM	mM	mM	mM	
HEPES	H0887-100ML	Sigma-Aldrich	50	50	50	50	pH = 7.6
Ammonium chloride	09718-250G	Sigma-Aldrich	1000				
Magnesium chloride	63020-1L	Honeywell Fluka	10	10	10	10	
Potassium chloride	P5405-1KG	Sigma-Aldrich		100	100	100	
Imidasol	I2399	Sigma-Aldrich		500			pH = 7
Glycerol	G7757-1L	Sigma-Aldrich				30%	
β-mercaptoethanol	M6250-100ML	Sigma-Aldrich	7	7	7	7	

Ribosome purification buffers

Compound	Catalog number	Company	Buffer C	Buffer D	Ribosome buffer	Note
			mM	mM	mM	
HEPES	H0887-100ML	Sigma-Aldrich			20	
Tris-HCl	BP152-500	Fisher	20	20		pH = 7.6
Magnesium acetate	M0631	Sigma-Aldrich			6	
Magnesium chloride	63020-1L	Honeywell Fluka	10	10		
Potassium chloride	P5405-1KG	Sigma-Aldrich	150	150	30	
Ammonium chloride	09718-250G	Sigma-Aldrich	30	30		
Imidasol	I2399	Sigma-Aldrich		150		pH = 7
β-mercaptoethanol	M6250-100ML	Sigma-Aldrich	7	7	7	

Energy solution

Compound	Catalog number	Company	Concentration in reaction	Concentration in subset (2.5x)	Units
Amino acids	LAA21-1KT	Sigma-Aldrich	0.3	0.75	mM
Magnesium acetate	M0631	Sigma-Aldrich	11.8	29.5	mM
Potassium glutamate	49601	Sigma-Aldrich	100	250	mM
TCEP	646547	SantaCruz Biotech	1	2.5	mM
ATP	R0481	ThermoFisher	2	5	mM
GTP	R0481	ThermoFisher	2	5	mM
CTP	R0481	ThermoFisher	1	2.5	mM
UTP	R0481	ThermoFisher	1	2.5	mM
tRNA	10109541001	Roche	52	130	U_{A260}/mL
Creatine phosphate	27920	Sigma-Aldrich	20	50	mM
Folinic acid	PHR1541	Sigma-Aldrich	0.02	0.05	mM
Spermidine	S2626	Sigma-Aldrich	2	5	mM
HEPES	H0887-100ML	Sigma-Aldrich	50	125	mM

References

[1] Eyal Karzbrun, Jonghyeon Shin, Roy H. Bar-Ziv, and Vincent Noireaux. Coarse-grained dynamics of protein synthesis in a cell-free system. *Physical Review Letters*, 106(4):048104, 2011.

[2] Tobias Stögbauer, Lukas Windhager, Ralf Zimmer, and Joachim O. Rädler. Experiment and mathematical modeling of gene expression dynamics in a cell-free system. *Integrative Biology*, 4(5):494–501, 2012.

[3] Zoltán A. Tuza, Vipul Singhal, Jongmin Kim, and Richard M. Murray. An in silico modeling toolbox for rapid prototyping of circuits in a biomolecular "breadboard" system. *52nd IEEE Conference on Decision and Control*, 2013.

[4] Alexander Nieß, Jurek Failmezger, Maike Kuschel, Martin Siemann-Herzberg, and Ralf Takors. Experimentally Validated Model Enables Debottlenecking of in Vitro Protein Synthesis and Identifies a Control Shift under in Vivo Conditions. *ACS Synthetic Biology*, 6(10):1913–1921, 2017.

[5] Fabio Mavelli, Roberto Marangoni, and Pasquale Stano. A Simple Protein Synthesis Model for the PURE System Operation. *Bulletin of Mathematical Biology*, 77(6):1185–1212, 2015.

[6] Anne Doerr, Elise de Reus, Pauline van Nies, Mischa van der Haar, Katy Wei, Johannes Kattan, Aljoscha Wahl, and Christophe Danelon. Modelling cell-free RNA and protein synthesis with minimal systems. *Physical Biology*, 16(2):025001, 2019.

[7] Tomoaki Matsuura, Naoki Tanimura, Kazufumi Hosoda, Tetsuya Yomo, and Yoshihiro Shimizu. Reaction dynamics analysis of a reconstituted Escherichia coli protein translation system by computational modeling. *Proceedings of the National Academy of Sciences*, 114(8):E1336–E1344, 2017.

[8] Nicholas Horvath, Michael Vilkhovoy, Joseph A. Wayman, Kara Calhoun, James Swartz, and Jeffrey D. Varner. Toward a genome scale sequence specific dynamic model of cell-free protein synthesis in Escherichia coli. *Metabolic Engineering Communications*, 10:e00113, 2020.

[9] Andrea Y. Weiße, Diego A. Oyarzún, Vincent Danos, and Peter S. Swain. Mechanistic links between cellular trade-offs, gene expression, and growth. *Proceedings of the National Academy of Sciences*, 112(9):E1038–E1047, March 2015.

[10] Kelly A. Underwood, James R. Swartz, and Joseph D. Puglisi. Quantitative polysome analysis identifies limitations in bacterial cell-free protein synthesis. *Biotechnology and Bioengineering*, 91(4):425–435, 2005.

[11] Dan Siegal-Gaskins, Zoltan A Tuza, Jongmin Kim, Vincent Noireaux, and Richard M Murray. Gene Circuit Performance Characterization and Resource Usage in a Cell-Free "Breadboard". *ACS Synthetic Biology*, 3(6):416–425, 2014.

[12] Jun Li, Chi Zhang, Poyi Huang, Erkin Kuru, Eliot T. C. Forster-Benson, Taibo Li, and George M. Church. Dissecting limiting factors of the Protein synthesis Using Recombinant Elements (PURE) system. *Translation*, 5(1):e1327006, 2017.

[13] Henrike Niederholtmeyer, Viktoria Stepanova, and Sebastian J. Maerkl. Implementation of cell-free biological networks at steady state. *Proceedings of the National Academy of Sciences*, 110(40):15985–15990, 2013.
