# Supplementary material for: A partially self-regenerating synthetic cell
Source: Nat Commun. 2020 Dec 11;11:6340. doi: 10.1038/s41467-020-20180-6 (PMC7733450; doi:10.1038/s41467-020-20180-6)
Supplement: Supplementary file 3 — Description of Additional Supplementary Files [file 41467_2020_20180_MOESM3_ESM.pdf]

Title: Supplementary Movie 1

Description: Illustrative microfluidic reactor loading sequence.
