# Supplementary material for: A partially self-regenerating synthetic cell
Source: Nat Commun. 2020 Dec 11;11:6340. doi: 10.1038/s41467-020-20180-6 (PMC7733450; doi:10.1038/s41467-020-20180-6)
Supplement: Supplementary file 5 — Reporting Summary [file 41467_2020_20180_MOESM5_ESM.pdf]

nature research

Corresponding author(s): Sebastian J. Maerkl

Last updated by author(s): Nov 11, 2020

Reporting Summary

Nature Research wishes to improve the reproducibility of the work that we publish. This form provides structure for consistency and transparency in reporting. For further information on Nature Research policies, see our Editorial Policies and the Editorial Policy Checklist.

Statistics

For all statistical analyses, confirm that the following items are present in the figure legend, table legend, main text, or Methods section.

n/a	Confirmed	
☐	☒	The exact sample size (n) for each experimental group/condition, given as a discrete number and unit of measurement
☐	☒	A statement on whether measurements were taken from distinct samples or whether the same sample was measured repeatedly
☒	☐	The statistical test(s) used AND whether they are one- or two-sided<br>*Only common tests should be described solely by name; describe more complex techniques in the Methods section.*
☒	☐	A description of all covariates tested
☐	☒	A description of any assumptions or corrections, such as tests of normality and adjustment for multiple comparisons
☐	☒	A full description of the statistical parameters including central tendency (e.g. means) or other basic estimates (e.g. regression coefficient) AND variation (e.g. standard deviation) or associated estimates of uncertainty (e.g. confidence intervals)
☒	☐	For null hypothesis testing, the test statistic (e.g. F, t, r) with confidence intervals, effect sizes, degrees of freedom and P value noted<br>*Give P values as exact values whenever suitable.*
☒	☐	For Bayesian analysis, information on the choice of priors and Markov chain Monte Carlo settings
☒	☐	For hierarchical and complex designs, identification of the appropriate level for tests and full reporting of outcomes
☒	☐	Estimates of effect sizes (e.g. Cohen's d, Pearson's r), indicating how they were calculated

Our web collection on statistics for biologists contains articles on many of the points above.

Software and code

Policy information about availability of computer code

Data collection	Ti2 Eclipse Inverted Microscope (Nikon), Orca-Flash 4.0 V3 Digital CMOS Camera (Hamamatsu), Labview (2018), Matlab (2019), Nanodrop 1000, Synergy MX Gen5 Biotech
Data analysis	Python 3.7.3, Julia 1.4.2 (code available on GitHub: https://github.com/lbnc-epfl), DifferentialEquations.jl=6.15.0, Interpolations.jl=0.12.10, Sundials.jl=4.2.5, ImageJ 1.52p, Numbers 10.1,

For manuscripts utilizing custom algorithms or software that are central to the research but not yet described in published literature, software must be made available to editors and reviewers. We strongly encourage code deposition in a community repository (e.g. GitHub). See the Nature Research guidelines for submitting code & software for further information.

Data

Policy information about availability of data

All manuscripts must include a data availability statement. This statement should provide the following information, where applicable:
- Accession codes, unique identifiers, or web links for publicly available datasets
- A list of figures that have associated raw data
- A description of any restrictions on data availability

All data supporting the findings of this study are available within the article and its supplementary information files or from the corresponding author upon reasonable request. Source data are provided with this paper. The source data underlying Fig. 2b, 3b, 4b and Supplementary Figs. 3, 4, 5, and 24 are provided as a Source Data file. The source data underlying Fig. 2c-d, 3c–d, 4a and 4c and Supplementary Figs. 6, 8, 20 and 21 are provided in a second separate Source Data file.

Field-specific reporting

Please select the one below that is the best fit for your research. If you are not sure, read the appropriate sections before making your selection.

☒ Life sciences ☐ Behavioural & social sciences ☐ Ecological, evolutionary & environmental sciences

For a reference copy of the document with all sections, see nature.com/documents/nr-reporting-summary-flat.pdf

Life sciences study design

All studies must disclose on these points even when the disclosure is negative.

Sample size	The sample sizes were chosen based on other microfluidic and cell-free studies reported in the literature ((Niederholtmeyer, 2013), (Swank, 2019), (Lavickova, 2019)). A typical experiment was repeated in two independent microfluidic chip compartments for each condition. No statistical methods were used to determine the sample size.
Data exclusions	A small fraction of data from microfluidic experiments was excluded if a malfunction of chip occurred due to delamination or precipitation in a given chemostat.
Replication	Attempts of replication were successful on different days with different microfluidic chips and DNA preparations. A typical experiment was repeated in two independent microfluidic chip compartments for each condition. Plate-reader experiments were performed in technical replicates of at least two; the number of replicates are specified in the relevant Figure legends.
Randomization	Not applicable to this study because group allocation was not carried out.
Blinding	Not applicable to this study because group allocation was not carried out.

Reporting for specific materials, systems and methods

We require information from authors about some types of materials, experimental systems and methods used in many studies. Here, indicate whether each material, system or method listed is relevant to your study. If you are not sure if a list item applies to your research, read the appropriate section before selecting a response.

Materials & experimental systems

n/a	Involved in the study	
☒	☐	Antibodies
☒	☐	Eukaryotic cell lines
☒	☐	Palaeontology and archaeology
☒	☐	Animals and other organisms
☒	☐	Human research participants
☒	☐	Clinical data
☒	☐	Dual use research of concern

Methods

n/a	Involved in the study	
☒	☐	ChIP-seq
☒	☐	Flow cytometry
☒	☐	MRI-based neuroimaging
